# Comparative lipidome study of maternal plasma, milk, and lamb plasma in sheep

**DOI:** 10.1038/s41598-024-58116-5

**Published:** 2024-03-28

**Authors:** Soundara Viveka Thangaraj, Adel Ghnenis, Brooke Pallas, Arpita Kalla Vyas, Brigid Gregg, Vasantha Padmanabhan

**Affiliations:** 1 Department of Pediatrics, University of Michigan, 7510 MSRB 1, 1500 W. Medical Center Drive, Ann Arbor, MI 48109 USA; 2 Unit for Laboratory Animal Medicine, University of Michigan, Ann Arbor, MI USA; 3 Department of Pediatrics, Washington University School of Medicine in St. Louis, St. Louis, MO USA

**Keywords:** Biochemistry, Developmental biology, Medical research

## Abstract

Lipids play a critical role in neonate development and breastmilk is the newborn’s major source of lipids. Milk lipids directly influence the neonate plasma lipid profile. The milk lipidome is dynamic, influenced by maternal factors and related to the maternal plasma lipidome. The close inter-relationship between the maternal plasma, milk and neonate plasma lipidomes is critical to understanding maternal-child health and nutrition. In this exploratory study, lipidomes of blood and breast milk from Suffolk sheep and matched lamb blood (n = 13), were profiled on day 34 post birth by untargeted mass spectrometry. Comparative multivariate analysis of the three matrices identified distinct differences in lipids and class of lipids amongst them. Paired analysis identified 346 differential lipids (DL) and 31 correlated lipids (CL) in maternal plasma and milk, 340 DL and 32 CL in lamb plasma and milk and 295 DL and 16 CL in maternal plasma and lamb plasma. Conversion of phosphatidic acid to phosphatidyl inositol was the most active pathway in lamb plasma compared to maternal plasma. This exploratory study illustrates the partitioning of lipids across maternal plasma, milk and lamb plasma and the dynamic relationship between them, reiterating the need to study these three matrices as one biological system.

## Introduction

Breastmilk is the primary source of nutrition for infants and offers multiple health benefits to infants and mothers. The World Health Organization (WHO) and American Academy of Pediatrics recommend exclusive breastfeeding for the first 6 months of life^1^. Milk is a complex and dynamic matrix of proteins, lipids, carbohydrates and other bioactive compounds^2^. Among these components, lipids are diverse biological molecules that function as a primary energy source for infants and play a critical role in several cellular and molecular functions. Lipids present in milk play regulatory roles in neonatal intestinal development^3^, infant brain development^4^, cognitive function^5^ and protect against metabolic, cardiovascular and other non-communicable diseases in adulthood^6–8^. A thorough understanding of the lipidome of milk is essential to identify the specific lipids that play key roles in infant growth and development.

Milk is the major source of lipids for neonates and hence the lipid profile of the offspring is dependent on the milk lipidome. This becomes even more important in premature infants who have not yet built their adipose tissue stores. Increasing levels of fatty acids like docosahexaenoic acid (DHA) in milk resulted in their increased levels in infant plasma^9^ and milk fatty acid (FA) composition influences infant serum lyso phosphatidylcholine^10^. The lipidome of milk is dynamic and changes through a 24-h cycle^11^ and across the lactation period^12^. It is influenced by maternal factors like diet, age, body mass index and stress^13,14^. The maternal plasma lipidome is a direct reflection of the maternal nutrient status and hence there exists a relationship between the maternal plasma and milk lipidomes. The maternal circulating FA is the major source of many FA in milk^15^ and the influence of maternal plasma FA status on the milk FA status is well established^16,17^. Similarly, studies have established a correlation between maternal plasma and cord blood lipidomes^18^ and between maternal plasma and neonate plasma phospholipids^16^, indicating the infant plasma lipid profile to be a function of both maternal plasma and milk lipid profiles. These studies collectively point towards the inter-relationship between maternal plasma, milk, and infant plasma lipid profiles and a possible mediating role played by milk in the transfer of lipids from the mother to infant in the postnatal period.

In addition to the maternal environment during gestation, the partitioning of lipids at the mammary gland and their assimilation in the infant are important factors contributing to child health. Milk interacts with maternal factors and influences infant development in parallel and an in-depth analysis of the associations between the maternal plasma, milk and infant plasma could further substantiate the mother-milk—infant as one biological system^19,20^. Studies have investigated specific phospholipids in maternal plasma, milk and neonate plasma and found correlations between them. For example, supplementation of breastfeeding mothers with DHA resulted in correlation between the levels of DHA in maternal plasma and milk^16^ and also between their levels in milk and infant plasma phospholipids^21^. However, these overly simplistic approaches only indicate the contribution of individual lipid species and do not fully reflect the dynamic changes that occur in the lipidome of the three compartments. To our knowledge, only Furse et al.^22^, have investigated the relationship between the lipidome of maternal plasma and lipid plasma through milk in a small cohort of human mother–infant pairs. Filling the gap in research on the relationships between maternal plasma, milk, and lamb plasma lipidome is an important step in maternal-child health and nutrition. To fill this gap and understand the transfer of lipids between the three matrices and identify relevant changes in lipid metabolism pathways in each of the three compartments we compared the lipidomes of maternal plasma, milk and offspring plasma in a sheep model. With lipids playing a vital role in infant growth and development and the lipid profile of the maternal plasma, milk and lamb plasma influencing each other, this exploratory study aimed to identify the differences in the lipidome between the maternal plasma, milk and lamb plasma and examine if it is reflective of the lipid transfer between compartments and the lipid metabolism occurring during milk production in mother and milk assimilation in lambs.

## Results

### Overview of plasma and milk lipidomics

**Figure 1:**
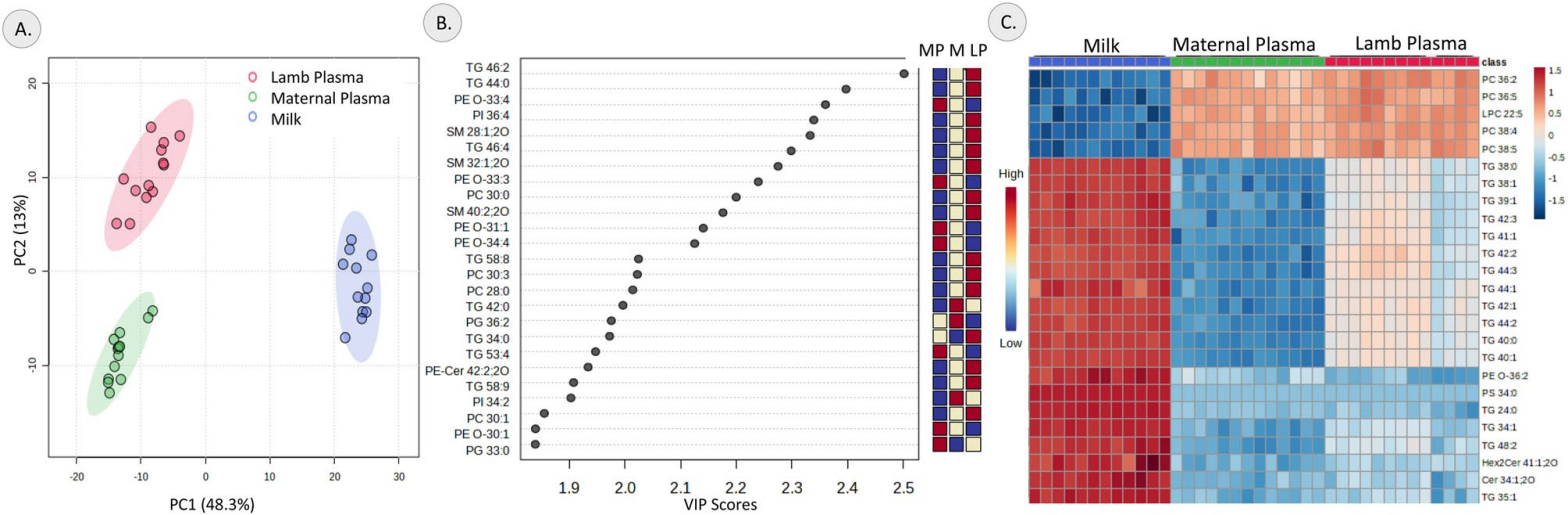
Comparison of maternal plasma, milk and lamb plasma lipidome. (**A**) Principal component analysis score plot based on lipids from maternal plasma, lamb plasma and milk, (**B**) Variables of importance in projection (VIP) plot based on multivariate partial least squares-discriminant analysis between maternal plasma (MP), lamb plasma (LP) and milk (M) (**C**) Heatmap of the top 25 significant differential lipids based on univariate ANOVA analysis between maternal plasma, lamb plasma and milk.

A total of 912 lipid species representing 37 lipid classes was profiled in the maternal and lamb plasma samples; 862 lipid species representing 37 lipid classes were identified in the milk samples. The different class of lipids present in the milk, maternal and lamb plasma samples are listed in supplementary table S1. For the comparative analysis involving both plasma and milk, only the 567 lipid species that were identified in both plasma and milk samples were used for the analysis. Principal component analysis of the three samples identified 2 major components with principal component 1 (PC1) explaining 48.3% of the variance and the principal component 2 (PC2) explaining 13% of the variance (Fig. 1A). Partial least squares-discriminant (multivariate) analysis of maternal plasma, lamb plasma and milk confirmed the distinction between them and the lipids that contributed most to the differences between the three samples are listed in Fig. 1B. The top 25 differential lipids identified by univariate analysis are represented as a heatmap in Fig. 1C. The plasma samples contained 245 lipid species that were absent in the milk samples (Supplementary Table S2) and 217 lipid species present exclusively in the milk samples (Supplementary table S3). Several fatty acyl esters of hydroxy fatty acid (FAHFA) lipids were found exclusively in milk compared to plasma (Supplementary table S2) while lipids of the lyso N-acylphosphatidylethanolamine (LNAPE) subclass were exclusively detected in plasma (Supplementary table S3).

### Milk lipidome distinct from maternal plasma lipidome

**Figure 2:**
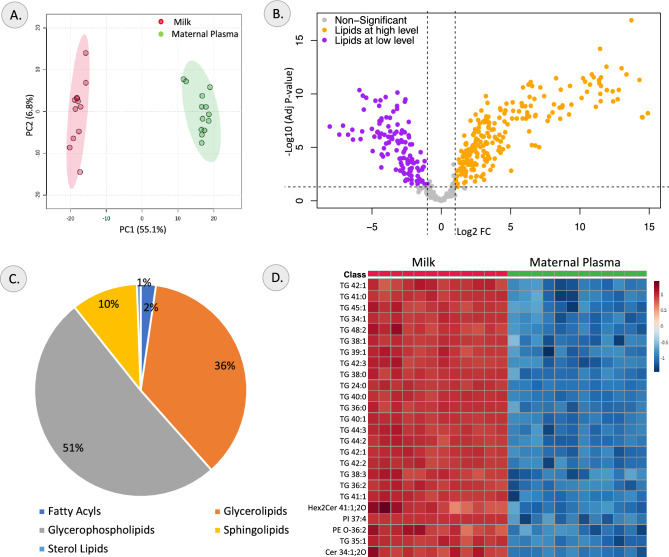
Changes in lipidome profile between milk and maternal plasma. (**A**) PCA Score plot, (**B**) Volcano Plot of differentially abundant lipids, (**C**) Pie-chart showing lipid class distribution of differential lipids, and (**D**) Heatmap of top differential lipids identified in comparison between milk and maternal plasma samples.

The maternal plasma and the milk lipidomes were compared to understand the relative difference in the lipid species between the two samples. The PCA analysis showed a clear clustering of the maternal plasma and milk samples, with PC1 and PC2 explaining 62.2% and 6% of the variance, respectively (Fig. 2A). Similar clustering of the samples was also seen in the OPLS-DA model, which had a R^2^Y value of 0.99 and Q^2^ score of 0.99, indicating a robust model (Supplementary Figure S1A). The top 25 lipid species that contribute the most to the difference between the two samples is represented by the variables of importance in projection (VIP) plot (Supplementary Figure S1B). A total of 346 differential lipid species were identified (Fig. 2B); 123 lipid species were present at lower concentrations and 223 lipid species were present at higher concentrations in the milk compared to the maternal plasma (Supplementary table S4). The lipids that showed lower relative abundance in milk primarily included lipids of the lyso-phosphatidylcholine (LPC), *N*-acyl lysophosphatidylethanolamine (LNAPE N-) and phosphatidylcholine (PC) with PC 38:4, PC 36:2, LPC 22:5, PC 38:5 and PC 36:5 being the most significant lipids. The lipids that were most significantly elevated in milk were the triacylglycerol (TG) lipids TG 24:0, TG 40:0, TG 36:0, TG 40:1 and TG 38:0. Most of the identified differential lipids were glycerophospholipids and glycerolipids as represented in the pie-chart showing classification of the differential lipids in Fig. 2C. The top 25 differential lipids in milk compared to maternal plasma are represented as a heatmap in Fig. 2D.

### Milk lipidome distinct from lamb plasma lipidome

**Figure 3:**
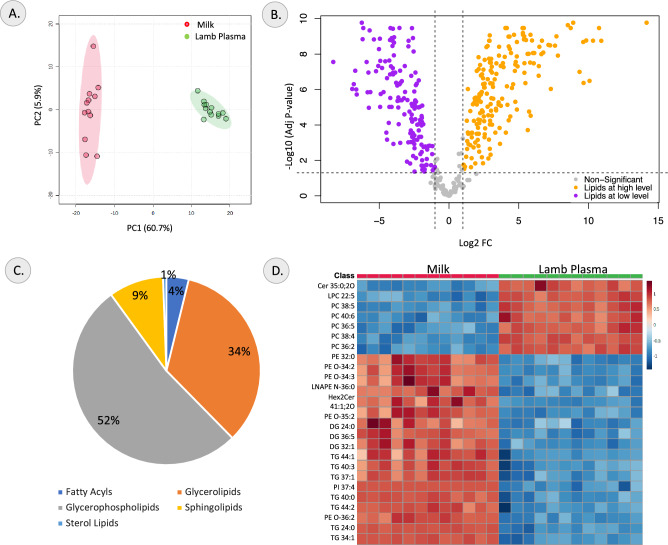
Changes in lipidome profile between milk and lamb plasma. (**A**) PCA Score plot, (**B**) Volcano plot of differentially abundant lipids, (**C**) Pie-chart showing lipid class distribution of differential lipids, and (**D**) Heatmap of top differential lipids identified in comparison between milk and lamb plasma samples.

The lamb plasma and the milk lipidomes were compared to understand the relative difference in the lipid species between the two samples. The PCA analysis demonstrated that PC1 and PC2 explained 60.6% and 6% of the variance, respectively and showed clear clustering based on the sample type (Fig. 3A). The generated OPLS-DA model had a R^2^Y value and Q^2^ score of 0.99, representing a robust model represented in Supplementary Figure S1C. The top 25 lipid species that contribute the most to the difference between the two samples is represented by the VIP plot (Supplementary Figure S1D). A total of 340 differential lipid species (Supplementary table S5)—149 lipids had significantly lower relative abundance and 191 lipids had significantly higher relative abundance in milk were identified and represented in the volcano plot in Fig. 3B. The lipids that were most significantly less abundant in milk were PC 38:4, PC 36:2, LPC 22:5, PC 38:5 and Cer 35:0;2O. The lipid groups that showed higher relative abundance in milk were primarily TGs and diacylglycerols (DG) with TG 24:0, TG 40:3, PE O-36:2, TG 34:1 and TG 37:1 being the most significant. Most of the identified differential lipids were glycerophospholipids and glycerolipids as represented in the pie-chart showing lipid class of the differential lipids in Fig. 3C. The top 25 differential lipids in milk compared to lamb plasma are represented as a heatmap in Fig. 3D.

### Maternal plasma lipidome differs from lamb plasma lipidome

**Figure 4:**
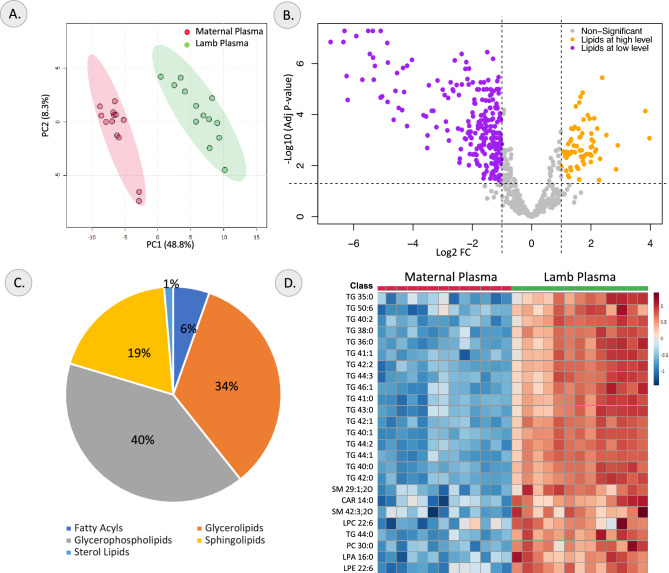
Changes in lipidome profile between maternal plasma and lamb plasma. (**A**) PCA Score plot, (**B**) Volcano plot of differentially abundant lipids, (**C**) Pie-chart showing lipid class distribution of differential lipids and (**D**) Heatmap of top differential lipids identified in comparison between maternal plasma and lamb plasma samples.

The maternal plasma and lamb plasma lipidomes were compared to gain insights into the transfer of lipids from ewes to lambs via milk. The PCA analysis showed PC1 and PC2 explained 48.8% and 8.3% of the variance, respectively (Fig. 4A). A supervised OPLS-DA analysis showed a clear clustering of the maternal and lamb plasma samples. The generated OPLS-DA model had a R^2^Y value of 0.94 and Q^2^ score of 0.91, representing a robust model (Supplementary Figure S1E). The top 25 lipid species that contribute the most to the difference between the two samples is represented by the VIP plot (Supplementary Figure S1F).

A total of 295 lipid species that showed differential abundance between maternal and lamb plasma are represented in the volcano plot in Fig. 4B and listed in Supplementary Table S6. Of these, 229 lipid species, primarily TG lipids like TG 40:0, TG 40:1, TG 44:1, TG 44:2 and TG 42:1 were present at lower concentrations and 66 lipid species including PE O-33:3, PE O-33:2, PE O-34:5, TG 53:4 and PE 33:0 were present at higher concentrations in the maternal plasma compared to the lamb plasma. Lipids of the PE O- and LNAPE N- classes showed higher relative abundance in the maternal plasma while several acylcarnitines (CAR), ceramides (Cer), DG, hexosylceramide (HexCer), phosphatidylinositol (PI) and sphingomyelin (SM) were predominantly higher in the lamb plasma. Most of the identified differential lipids were glycerophospholipids and glycerolipids as represented in the pie-chart showing lipid class of the differential lipids in Fig. 4C. The top 25 differential lipids are represented as a heatmap in Fig. 4D.

### Correlation of lipid relative abundance across the plasma and milk lipidome

**Figure 5:**
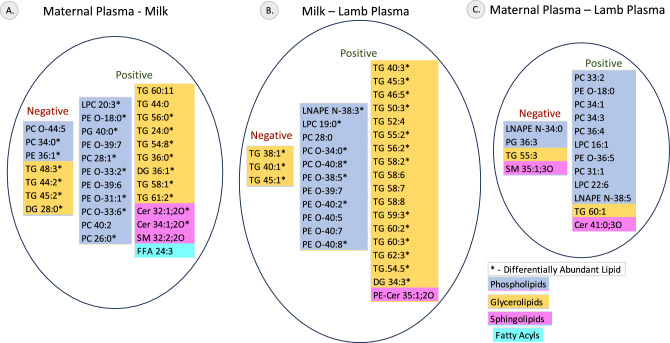
Lipids correlated between maternal plasma, milk and lamb plasma. Lipids identified to be significantly correlated between (**A**) maternal plasma and milk, (**B**) milk and lamb plasma, (**C**) maternal plasma and lamb plasma are indicated. Lipid subclass is color-coded according to the scale. Lipids that were both correlated and identified as differential lipids are indicated with an *.

Correlation analysis identified relative abundance of several lipids to be correlated between the different samples. The relative abundance of 31 lipids were correlated between maternal plasma and milk and relative abundance of 32 lipids were correlated between milk and lamb plasma. In contrast, the maternal plasma and lamb plasma had only 16 lipids to be correlated between them. The relative abundance of PE O-18:0 was correlated between maternal plasma and lamb plasma and also between maternal plasma and milk. The correlated lipids, their class and direction of correlation are illustrated in Fig. 5 and listed in Supplementary table S7. Several of these correlated lipids were also identified to be differentially abundant between the samples.

### Lipid class level differences

**Figure 6:**
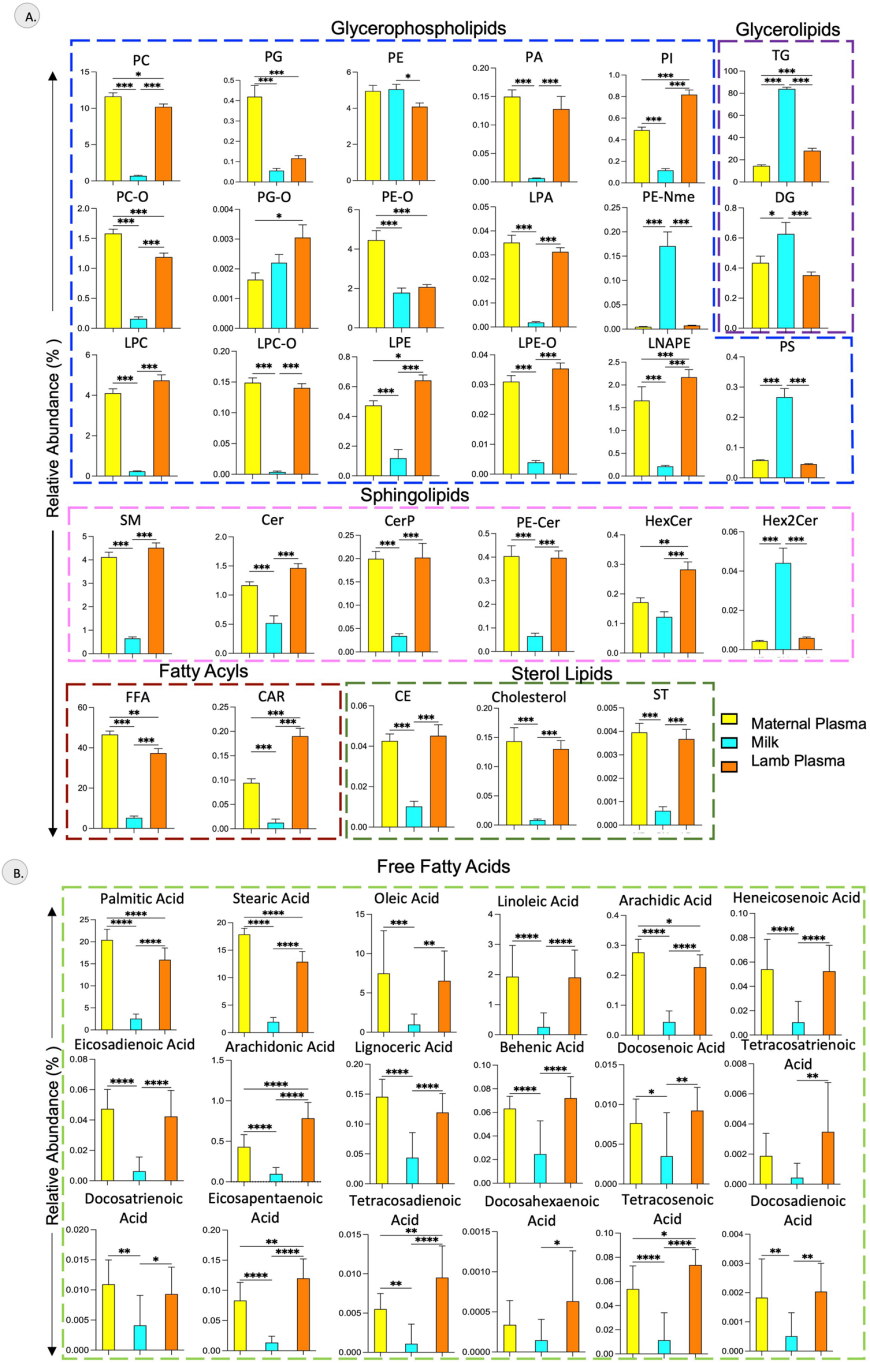
Differences in the distribution of lipid classes and fatty acids in maternal plasma, milk and lamb plasma. (**A**) Bar graph represents relative abundance (%) of different classes of lipids between the maternal plasma, milk and lamb plasma, (**B**) Bar graph represents relative abundance (%) of different fatty acids between the maternal plasma, milk and lamb plasma, expressed as percentage (n = 13/group). *P < 0.05 **P < 0.01, *** *P* < 0.001 by two-way ANOVA with Tukey’s multiple comparison test.

The class level differences in the distribution of lipids in maternal plasma, milk and lamb plasma are illustrated in Fig. 6A. The free fatty acids (FFA), acylcarnitines (CAR), phosphatidylcholine (PC), ether-linked phosphatidylcholine (PC-O), phosphatidylinositol (PI), lysophosphatidylethanolamine (LPE) triacylglycerol (TG), and N-acyl-lysophosphatidylethanolamine (LNAPE) showed distinct differences in their relative abundance between maternal plasma and milk, milk and lamb plasma as well as maternal plasma and lamb plasma. The relative abundance of phosphatidylglycerol (PG), ether-linked phosphatidylglycerol (PG-O), ether-linked phosphatidylethanolamine (PE-O) and hexosylceramide (HexCer) was different between the maternal and lamb plasma. The relative abundance of the sterol lipids- cholesterol, cholesteryl ester (CE) and brassicasterol (ST), the ceramides—sphingomyelin (SM), ceramides (Cer), ceramide phosphates (CerP), ceramide phosphoethanolamine (PE-Cer) were higher in both maternal and lamb plasma compared to milk. The relative abundance of TG, diacylglycerol (DG), phosphatidylserine (PS), and dihexosylceramide (Hex2Cer) were higher in the milk samples compared to both maternal and lamb plasma samples. The relative abundance of ether-linked TG (TG-O) and fatty acyl esters of hydroxy fatty acid (FAHFA) were similar across maternal plasma, milk and lamb plasma.

Taking a closer look at the relative abundance of the fatty acids, that play a critical role in infant development, the relative abundance of palmitic acid, stearic acid, arachidic acid, arachidonic acid, eicosapentaenoic acid, tetracosanoic acid and tetracosadienoic acid were significantly different between maternal plasma, milk and lamb plasma (Fig. 6B). All the fatty acids showed higher relative abundance in the plasma as compared to milk. The relative abundance of arachidonic acid, eiscosapentaenoic acid, behenic acid, tetracosanoic acid and tetracosadienoic acid was higher in the lamb plasma compared to maternal plasma. The relative abundance of oleic acid, linoleic acid, heneicosenoic acid, eicosadienoic acid, behenic acid, docosenoic acid, docosadienoic acid, docosatrienoic acid and lignoceric acid were similar between maternal and lamb plasma but lower in milk.

### Correlation of lipid classes across the plasma and milk lipidome

**Figure 7:**
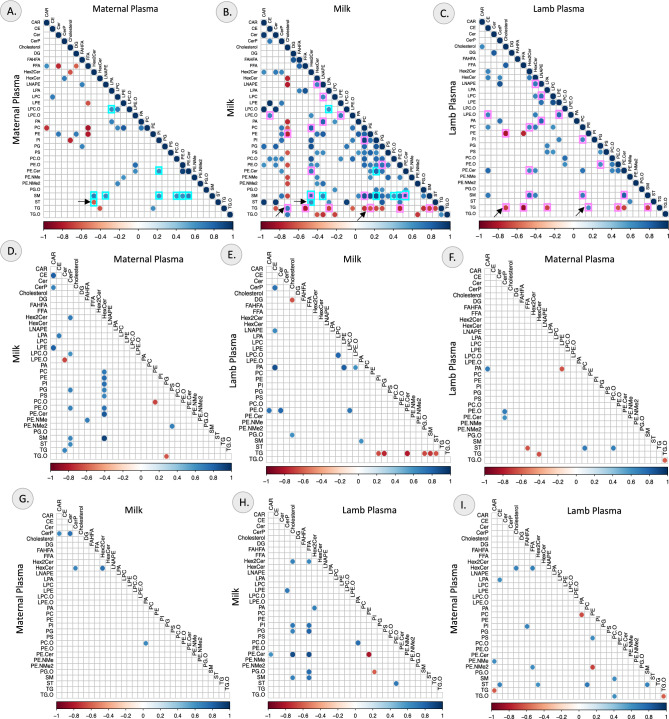
Correlations between lipid classes in maternal plasma, milk and lamb plasma. Spearman correlation matrix between lipid classes within (**A**) Maternal plasma, (**B**) Milk and (**C**) Lamb plasma; correlations that are seen in both maternal plasma and milk are denoted by cyan boxes and those seen in both milk and lamb plasma are denoted by pink boxes; correlations with a change in directionality between the samples are indicated by black arrows. Spearman correlation matrix between lipid classes between (**D**) Milk and Maternal plasma, (**E**) Lamb plasma and Milk, (**F**) Lamb plasma and Maternal plasma, (**G**) Maternal plasma and Milk, (**H**) Milk and Lamb plasma and (**I**) Maternal plasma and lamb plasma. Pairwise, unidirectional correlations are indicated by the color spectrum with positive correlations represented by blue color and negative correlations represented by red color. The strength of correlation is indicated by the color intensity. Statistically significant correlations with *P* < 0.05 and correlation coefficient >  ± 0.6 are represented.

The correlation between the different lipid classes within the maternal plasma, milk and lamb plasma are represented in Fig. 7A, B and C. The milk samples showed maximum correlation between the different lipid classes compared to other sample types. The correlation between several lipid classes in the lamb plasma were preserved in the milk but fewer common correlations between maternal plasma and milk. While maternal plasma showed a negative correlation between sterols and Hex2Cers, in milk the two lipid classes showed a positive correlation. Similarly, TG showed a negative correlation with cholesterol and a positive correlation with phosphatidylethanolamine (PE) in lamb plasma, but the direction of correlation was reversed in milk. Pairwise correlation matrix of the lipid classes between maternal plasma, lamb plasma and milk are indicated in the correlation plots in Fig. 7D–I. Multiple lipid classes in milk were positively correlated with HexCer lipid class in the maternal plasma samples. TG in lamb plasma was negatively correlated with several lipid classes in milk.

### Lipid conversion pathways activated across plasma and milk samples

**Figure 8:**
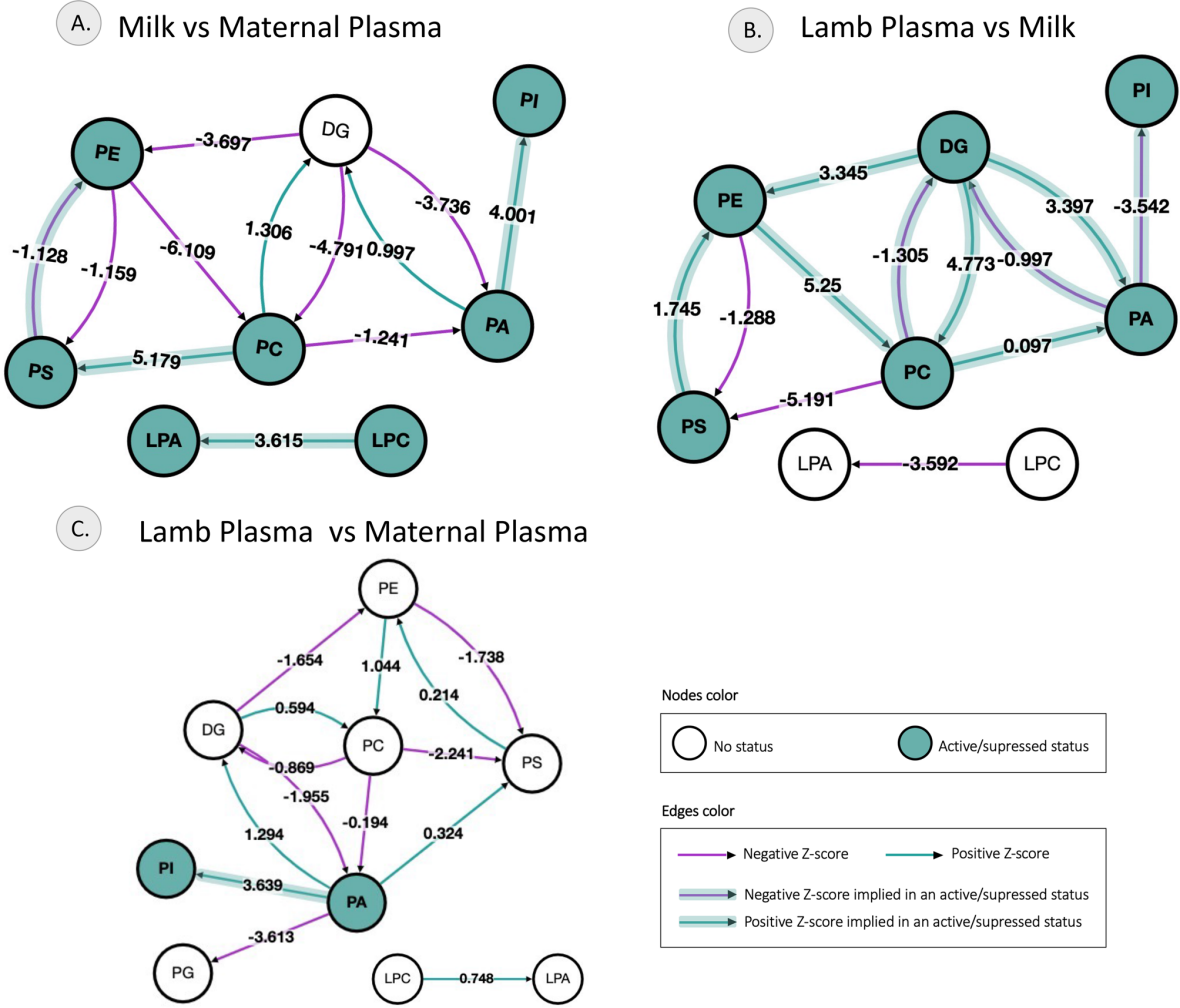
Active Pathways identified in maternal plasma, milk and lamb plasma based on lipid classes. Lipid network graph showing pathways activated in (**A**) Milk compared to maternal plasma (**B**) Lamb plasma compared to milk and (**C**) Lamb plasma compared to maternal plasma. Green nodes correspond to active lipids and green shaded arrows to active pathways. Reactions with a positive Z score have green arrows while negative Z scores are colored purple.

The reaction pathway prediction identified the most active lipid conversions in each sample compared to another sample type based on the differential lipids. The most active conversion pathways in milk compared to maternal plasma were PC to PS, PA to PI and LPC to LPA. In the lamb plasma, the most active conversion pathways compared to milk were DG to PA, PC and PE, PC to PE and PE to PC, while the conversion from PC to PS, PA to PI and LPC to LPA were suppressed. Comparison of the lamb plasma with the maternal plasma indicated the most active conversion pathway to be PA to PI. The conversion pathways are illustrated in Fig. 8 and the z-scores are listed in Supplementary table S8.

## Discussion

Lipid profiling of maternal plasma, milk and lamb plasma in sheep identified distinct differences and associations between the lipidome of maternal plasma, milk and lamb plasma samples. Although the milk lipidome is distinct from both maternal and lamb plasma lipidomes, it is the primary mediator of the plasma lipidome of lambs that are dependent on the milk for their dietary needs. Similarly, the maternal plasma is the primary source of lipids for the milk and plays an important role in deciding the lipid content of lamb plasma through milk. It is the metabolism of fatty acids carried by the milk lipids, that determines the composition of lamb plasma lipids. The lipidome can be considered a two-dimensional matrix constituted by individual lipid species and the lipid classes, since changes in the level of one lipid species may be compensated by another lipid species belonging to the same lipid class. Similarly, perturbation of a metabolic pathway can impact the levels of all lipids involved in the pathway across all lipid classes. We have identified individual lipid species and lipid subclasses that are differentially abundant and correlations between the maternal plasma, lamb plasma and milk, which gives insights into the metabolism of lipids across these matrices.

The lipidome of milk was distinctly different from both maternal and lamb plasma. The differentially abundant lipids that showed higher relative abundance in the milk compared to maternal plasma were mainly TGs, similar to humans where maternal plasma is known to have a lower TG content than milk^23^. TGs are an important source of energy for the growing lamb and provide more than 50% of the caloric intake^24^. Among the lipid classes, the DGs, TGs, PSs and Hex2Cers were the only class of lipids whose relative abundance was higher in milk than the maternal plasma or lamb plasma. PS is an important component of human brain that is implicated in neurotransmission and cognitive function^25^. Several FAHFA lipids were exclusively present in milk. This family of lipids has been associated with both maternal body mass index and metabolic outcomes, making them of interest as possible mediators of developmental programming^26,27^. Lower relative abundance of all the sterol lipids were observed in milk compared to maternal and lamb plasma but their relative abundance was similar between the two plasma samples. This is suggestive that intestinal sterol synthesis is a significant contributor to the lamb plasma sterols^28^ in addition to the sterols sourced from milk.

The differences in the lipid profile and the pattern of correlation between the lipid classes in maternal plasma and milk indicate the extensive remodeling of plasma lipids in the mammary gland with the mammary epithelial cells (MEC) preferentially enriching certain lipid species. MEC have a unique physiologic process by which they secrete lipids. Cytoplasmic lipid droplets mainly composed of a core of TG with a phospholipid monolayer are enveloped by the bilayer plasma membrane to create milk fat globules. The plasma membrane is primarily composed of cholesterol, PC, PE and SM^29,30^. The details of molecular events that govern milk production by MEC is not yet fully resolved. Enzymes responsible for de novo fatty acid synthesis and hydrolysis of TG are upregulated while those involved in beta-oxidation and TG storage are decreased during mammary gland development^31^. This establishes a flow of glycerol and fatty acids into milk lipid production. Fatty acids with a carbon backbone > 14 is not synthesized de novo by the gland but rather supplied from maternal circulation^32^. While the details of this process of milk lipid assembly and secretion need further investigation, these studies support the extensive assembly of TG by the mammary gland and could explain the increased relative abundance of TG and DG seen in milk compared to plasma samples.

When lambs suckle, the esophageal groove closes, and the milk by-passes the rumen as it gets shunted to the abomasum and then to the small intestine, where it is enzymatically broken, and the lipids enter the blood stream packaged into chylomicrons^33^. The correlation pattern between lipid classes was more similar between milk and lamb plasma, indicating the lipid transfer to the lamb through milk. The larger number of lipid-class correlations in milk may be due to shared biosynthetic pathway or co-regulation by cellular mechanisms. The altered direction of correlation of lipid classes across milk and plasma may be a function of the difference in lipid metabolism. The candidate lipid conversion pathways that are activated in lactation in this study allow for prediction of genes involved in the conversion process. Several of these candidate genes have been associated with milk fat yield in sheep (CDS1)^34^ and cows (ENPP2^35^, PTDSS1^36^), supporting the plausibility of these findings. The difference between lamb plasma and milk lipidomes is contributed by the digestive process in lambs^22^. The strong correlations between the relative abundance of several lipids in milk—maternal plasma and milk-lamb plasma indicate the close relationship between these three microenvironments, supporting the need to consider them as one biological system^20^. These findings have indeed been supported by previous work focused on the milk long-chain fatty acids across the three components of this system^37^. Serum levels of carnitines that were present at higher levels in lamb plasma, have been positively associated with postnatal growth and brain size in infants^38^.

Lipidome pathway analysis identified an activation of phosphatidic acid (PA) to phosphotidylinositols (PI) in lamb plasma compared to maternal plasma. PA is a key lipid mediator central to biosynthesis of lipids^39^ including PI, a cell membrane constituent playing a critical role in endoplasmic reticulum function and in postnatal development of newborns^40^. It is of interest that PI was also present at higher concentrations in lamb plasma compared to maternal plasma. The advent of lipidomics has led to the identification of several markers of infant obesity and metabolic disruption in milk and maternal plasma^41–45^. These studies that investigate the maternal plasma or milk in isolation, although informative, may not represent the complete picture. The inter-relationship between maternal plasma, milk and infant plasma lipids evidenced in our study, demonstrate the need to consider these three matrices which influence each other as one single system.

This is the first study, albeit exploratory, to describe the lipidome of maternal plasma, lamb plasma and milk from the same cohort of animals and only the second to report shotgun lipidomic across these biofluids during the lactation period. While the relative composition of long chain fatty acids during this life stage has been well described, a broader definition of the total lipids during lactation is just being created. Correlation between lipid classes in the different samples gives an integrated picture of lipid dynamics. We have assessed the partitioning of the lipids in parallel across these three matrices. This study is in a large animal model with a developmental ontology similar to humans. Neonate lambs maintained exclusively on milk, like in our study, show limited ruminal development due to the reflexive closure of the reticular groove^46^. As a result, the primary source of energy is intestinally absorbed nutrients that are blood borne, making our study translationally relevant to humans. Although the composition and lipid characteristics of sheep milk is distinctly different from human milk, the ratio of total PL, PC, PE and PS in sheep milk is similar to that in human milk^47^.

The timing of sample collection could have influenced the lipid profile. Since the lambs and ewes were housed together, the lambs had access to mother’s milk around the clock and fasting blood could not be collected for the lipidome analyses. If some of the lambs had fed just before blood collection and the others did not, it could have influenced the lamb plasma lipid profile. On the other hand, this reflects the typical feeding pattern of both humans and lambs. Prolonged fasting does not occur in the first month of life. Milk was collected from only one teat and could not be identified as foremilk or hindmilk. Parity, lamb sex and number of suckling lambs affect the fat content of milk^48^. These factors were not controlled for in our study which included a heterogenous mix of sheep with single, twin, and triple suckling lambs. The limited sample size was further affected by the distribution of lamb sex because of which sex differences could not be profiled in this study; replication of this study using a larger group of animals is warranted. Inherent biological differences in the gastrointestinal system of ruminants may result in significant differences in the metabolism of milk production^49^ and de novo synthesis of fatty acids^50^, which needs to be considered while translating the findings of this study to apply to human biology. The lipidome data presented here is relative quantitation data based on the use of select standards for each lipid class (untargeted) and cannot be compared to studies of absolute quantitation of lipids. The non-availability of accurate standards makes accurate quantification of milk lipids challenging^51^. Deciphering lipidomic data is challenging due to the heterogeneity of lipid classes, each containing numerous molecular variants and lipids readily undergo transformations between and with classes. So, studies like ours only give a snapshot of the lipid profile at the time of sample collection and may not reflect global lipid metabolism. Curated biochemistry-based resources are necessary for meaningful biological interpretation of the lipid correlations. A hypothesis-driven study was not proposed due to paucity of prior information or associated clinical/phenotypic endpoints, resulting in an exploratory hypothesis-generating observational study.

## Conclusion

This is the first proof of concept study to parallelly investigate the lipidome of maternal plasma, milk and lamb plasma. We have identified key lipids and lipid classes that are differentially present and correlated between the three matrices. Although there were several limitations to this study, the novel findings of this study illustrate the dynamic relationship that exists between the maternal plasma, milk and lamb plasma, that reiterates the need to study these three matrices as one biological system. This hypothesis-generating study sets the stage for further hypothesis-driven studies in the realm of lipid remodeling and transfer between mother and child and how the milk mediates this lipid transfer.

## Methods

### Animals

The present study is an offshoot of a parent study that examined the impact of gestational testosterone excess on offspring reproductive and metabolic outcomes as a model of polycystic ovarian syndrome phenotype. All experimental animal procedures were approved by the University of Michigan Animal Care and Use Committee and the methods were performed in accordance with the National Research Council’s Guide for the Care and Use of Laboratory Animals. Pregnant ewes were bought and maintained at the University of Michigan Sheep Research Facility (Ann Arbor, MI). This study is reported in accordance with ARRIVE guidelines^52^. Suffolk breed of sheep was used, and their maintenance and lambing were performed as described earlier^53^. The ewes were housed under natural photoperiod and were fed with a daily maintenance diet that consisted of 1.8 kg alfalfa/brome mix hay/ewe to prevent obesity. From 6 weeks before lambing, the ewes were fed with 1 kg shelled corn, 2.5 kg alfalfa hay/ewe. After birth, each lamb was given oral vitamin E and selenium injections as well as vaccinated for *Clostridium perfringens* types C and D and tetanus. Mother and lambs were housed individually for the first 3 days after birth and then group-housed with other mother-lamb pairs in a barn under natural photoperiod with a 60-W bulb in the lamb creep feed area at nights. All mother-lamb pairs that were available and where lambs were not provided supplemental bottle feed were used in this study; lambs that were bottle-fed were excluded. All the lambs, including the ones not involved in the experiment were kept with their mother and suckled milk freely. The lambs had access to the feed supplied to ewes and may have been nibbing on it.

### Prenatal treatment

In the parent study, pregnant ewes were randomly assigned to Control or prenatal testosterone (T) treatment groups. Prenatal T ewes were administered twice weekly intramuscular injections of 100 mg T propionate (Millipore Sigma, St. Louis, MO) suspended in corn oil, between gestational days 30 and 90. Control animals received the vehicle. We capitalized on maternal lamb pairs that were breast-feeding and available to profile lipidome of maternal plasma, lamb plasma and milk. Unfortunately, due to the small sample size, and mixed litter sizes we did not have enough power to detect the effect of testosterone treatment, to do a comparative control vs. prenatal T group study. We conducted an initial analysis and found prenatal T treatment did not result in changes in the maternal plasma, lamb plasma or milk lipids, as examined by univariate and multivariate analyses (Supplementary Figure S2). As such, the control and prenatal T animals were grouped together for the purpose of this study.

### Sample collection and processing

Samples of maternal plasma, lamb plasma and milk were collected into Vacutainer K2 EDTA tubes (BD, New Jersey, NJ) from each ewe-lamb pair (n = 13) on day 34 after birth in May 2021. Samples were obtained between 8:00 and 9:30 a.m. in the mornings. For each ewe-lamb pair, breast milk was obtained first, followed by blood sampling from the ewes and then from the lambs one after the other, on the same day. Prior to milk collection, teats were cleaned with wet gauze and milk was hand expressed into collection tube once milk flow was established. Collection of each set of samples was completed prior to moving to the next ewe-lamb pair. This period corresponds to the peak lactation period when ewes typically reach maximum milk production^54^. Blood was drawn by jugular venipuncture and plasma was harvested from collected blood samples and stored as aliquots at -80 °C. Untreated fresh milk (5 mL) was stored at − 80 °C until the time of analysis.

### Reagents and Internal Standards

High-performance liquid chromatography (HPLC) grade acetonitrile and dichloromethane and mass spectrometry-grade lipid standards were purchased from Sigma-Aldrich (St. Louis, MO), isopropanol (Optima—LC/MS grade) was purchased from Fisher (New Jersey, NJ) and methanol (LC–MS grade) was from J.T. Baker. The following mass spectrometry-grade lipid standards obtained from Sigma-Aldrich were used: 1-heptadecanoyl-2-hydroxy-*sn*-glycero-3-phosphocholine LPC (17:0/0:0), 1,2-diheptadecanoyl-sn-glycero-3-phosphocholine PC (17:0/17:0), 1,2-diheptadecanoyl-sn-glycero-3-phosphoethanolamine PE (17:0/17:0), 1,2-diheptadecanoyl-*sn*-glycero-3-phospho-l-serine (sodium salt) PS (17:0/17:0), N-heptadecanoyl-d-*erythro*-sphingosylphosphorylcholine 17:0 SM (d18:1/17:0), cholest-5-en-3ß-yl heptadecanoate 17:0 cholesteryl ester, 1-palmitoyl-2-oleoyl-*sn*-glycerol 16:0–18:1 DG, 1-heptadecanoyl-rac-glycerol 17:0 MG, 1,2,3-triheptadecanoyl-glycerol Triheptadecanoate 17:0TAG, *N*-heptadecanoyl-d-*erythro*-sphingosine C17 Ceramide (d18:1/17:0), 1,2-diheptadecanoyl-*sn*-glycero-3-phosphate (sodium salt) 17:0 PA, 1,2-diheptadecanoyl-*sn*-glycero-3-phospho-(1′-*rac*-glycerol) (sodium salt) 17:0 PG, 1-heptadecanoyl-2-(5Z,8Z,11Z,14Z-eicosatetraenoyl)-*sn*-glycero-3-phospho-(1′-myo-inositol) (ammonium salt) 17:0–20:4 PI, 1,3(d5)-dinonadecanoyl-2-hydroxy-glycerol DG d^5^-(19:0/0:0/19:0) and Glyceryl tri(palmitate-d^31^) TG d^31^.

### Extraction of lipid fraction

Lipids were extracted using a modified Bligh-Dyer method^55^. The extraction was carried using 2:2:2 volume ratio of water/methanol/dichloromethane at room temperature after spiking internal standards. The organic layer was collected and dried completely under nitrogen. Before mass spectrometry analysis, the dried lipid extract was resuspended in 100 μL of Buffer B (10:85:5 ACN/IPA/water) containing 10 mM ammonium acetate and subjected to LC–MS.

### Internal standards and quality controls

Quality control samples were prepared by pooling equal volumes of each sample and injected at the beginning and the end of each analysis and after every 10 sample injections to provide a measurement of the system’s stability and performance as well as reproducibility of the sample preparation method^56^. Two kinds of controls were used to monitor the sample preparation and mass spectrometry. To monitor instrument performance, 10 μL of a dried matrix-free mixture of the internal standards reconstituted in 100 μL of buffer B (85% IPA:10%ACN:5% H_2_O in 10 mM NH_4_OAc) was analyzed. As additional controls to monitor the profiling process, an equimolar mixture of the 13 authentic internal standards and a characterized pool of human plasma and test pool (a small aliquot from all plasma used in this study) (extracted in tandem with plasma samples) were analyzed along with the plasma samples. Each of these controls, were included several times into the randomization scheme such that samples preparation and analytical variability could be monitored constantly. The peak areas of internal standards were used to normalize the data. The QC samples were also used to remove technical outliers and lipid species that were detected below the lipid class-based lower limit of quantification.

### Mass spectrometry

Chromatographic separation was performed on Shimadzu CTO-20A Nexera X2 UHPLC (Canby, OR, USA) at the Metabolomics Core facility at the University of Michigan. For lipid separation, 5 μL of the lipid extract was injected onto a Waters Acquity HSS T3 column (Waters, Milford, MA) at a flow rate of 0.4 mL/min. Mass spectrometry data acquisition for each sample was performed in both positive and negative ionization modes using a TripleTOF 5600 equipped with a DuoSpray ion source (AB Sciex, Concord, Canada). Column effluent was directed to the ESI source and voltage was set to 5500 V for positive ionization and 4500 V for negative ionization mode. The curtain gas flow, nebulizer, and heater gas were set to 30, 40, and 45, respectively (arbitrary units). Acquisition of MS/MS spectra was controlled by the data dependent acquisition (DDA) function of the Analyst TF software (AB Sciex, Concord, Canada). Calibrations were performed at the initiation of each new batch or polarity change.

### Data processing

The raw data was converted to mgf data format using proteoWizard software^57^. The NIST MS PepSearch Program was used to search the converted files against LipidBlast^58,59^ libraries in batch mode. The MS/MS identification results from all the files were combined using an in-house software tool to create a library for quantification. All raw data files were searched against this library of identified lipids with mass and retention time using Multiquant 1.1.0.26^60^ (ABsciex, Concord, Canada). Quantification was done using MS1 data. The QC samples were also used to remove technical outliers and lipid species that were detected below the lipid class-based lower limit of quantification. All lipid levels are relative quantitative values normalized to class matching internal standards and these values are accurate since the ionization properties of individual lipid species within a lipid class are very similar. The average coefficient of variation of all the lipids detected in the study samples was 19%. All unknown lipids (80 in milk and 96 in plasma) were excluded from the analysis. All lipids within a class were added for each sample to get the total peak area for that sample and this was used for the class-level analysis of lipids.

### Statistical methods

Data from the positive and negative ion modes were combined and log-transformed for further analyses. Multivariate analyses were done using MetaboAnalyst 5.0^61^. The principal components, clusters, trends and outliers were identified and visualized using the unsupervised principal component analysis. One milk sample was identified as an outlier (indicated in Supplementary figure S3) and was removed from further analyses. The differences in lipid profiles of the different samples and the differential lipids were identified by Orthogonal projections to latent structure discriminant analysis (OPLS-DA). The models with Q^2^ and R^2^Y values > 0.5, as represented in the Q^2^/R^2^Y overview plot, indicated a stable and reliable model. Paired analyses were applied to the comparisons (maternal plasma vs lamb plasma, maternal plasma vs milk and lamb plasma vs milk). Volcano plots were generated using R. Differential lipids are identified as those with fold change > 2 and false discovery rate (FDR) adjusted *P* value < 0.05, identified by paired analysis. Relative abundance of lipid classes was calculated as a ratio of the sum of relative abundance of all lipid species within a lipid class to the sum of relative abundance of all lipids measured in that sample. Class analysis of lipids was carried out based on lipid groups and compared between samples by two-way ANOVA with a post-hoc Tukey’s test. Both univariate and multivariate statistical analysis was employed in this study as univariate test assesses the importance of each variable separately while the multivariate test involves the use of a statistical model to assess multiple variables simultaneously, factoring in the relationship between the variables.

CAR, carnitine; CE, cholesteryl ester; Cer, ceramide; DG, diacylglyceride; FAHFA, fatty acyl esters of hydroxy fatty acid; FFA, free fatty acid; Hex2Cer, dihexosylcermide; HexCer, hexosylceramide; LNAPE, *N*-acyl lysophosphatidylethanolamine; LPA, lyso-phosphatidic acid; LPC, lyso-phosphatidylcholine; LPC-O, ether-linked lyso-phosphatidyl-cholines; LPE, lyso-phosphatidylethanolamine; PA, phosphatidic acid; PC, phosphatidylcholine; PE, phosphatidylethanolamine; PE-O, ether-linked phosphatidylethanolamine; PG, phosphatidylglycerol; PG-O, ether-linked phosphatidylglycerol; PI, phosphatidylinositol; PS, phosphatidylserine; SM, sphingomyelin; TG, triacylglyceride, TG-O, ether-linked triacylglycerol.

### Correlation analysis

Correlation analyses were performed to examine the association of lipid and lipid class levels within and across the samples^62^ using the corrr package in R. Correlations with − 0.6 < r > 0.6 and *P* value < 0.05 were considered significant.

### Pathway analysis

The lipidome data was analyzed using the web-based tool, BioPAN^63^ to assess for lipid pathways based on available literature. Pathways with a positive and negative z-score are classed as active pathways and suppressed pathways, respectively. A positive z-score with active status implies it is a prominent pathway within this dataset.

### Supplementary Information

Supplementary Information.

## Data Availability

The raw data used to support the findings of this study are available on request from Dr. Vasantha Padmanabhan (vasantha@umich.edu).

## References

[1] Kramer MS, Kakuma R (2012). Optimal duration of exclusive breastfeeding. Cochrane Database Syst. Rev..

[2] Andreas NJ, Kampmann B, Mehring Le-Doare K (2015). Human breast milk: A review on its composition and bioactivity. Early Hum. Dev..

[3] Ramiro-Cortijo D (2020). Breast milk lipids and fatty acids in regulating neonatal intestinal development and protecting against intestinal injury. Nutrients.

[4] Schipper L, van Dijk G, van der Beek EM (2020). Milk lipid composition and structure: The relevance for infant brain development. OCL.

[5] Zheng L, Fleith M, Giuffrida F, O'Neill BV, Schneider N (2019). Dietary polar lipids and cognitive development: A narrative review. Adv. Nutr..

[6] Anto L, Warykas SW, Torres-Gonzalez M, Blesso CN (2020). Milk polar lipids: Underappreciated lipids with emerging health benefits. Nutrients.

[7] George AD (2022). The role of human milk lipids and lipid metabolites in protecting the infant against non-communicable disease. Int. J. Mol. Sci..

[8] van der Beek EM, Oosting A (2020). Nutritional programming in early life: The role of dietary lipid quality for future health. OCL.

[9] Gibson RA, Neumann MA, Makrides M (1997). Effect of increasing breast milk docosahexaenoic acid on plasma and erythrocyte phospholipid fatty acids and neural indices of exclusively breast fed infants. Eur. J. Clin. Nutr..

[10] Hellmuth C (2018). The impact of human breast milk components on the infant metabolism. PLoS One.

[11] George AD (2020). Untargeted lipidomics using liquid chromatography-ion mobility-mass spectrometry reveals novel triacylglycerides in human milk. Sci. Rep..

[12] Hewelt-Belka W, Garwolińska D, Młynarczyk M, Kot-Wasik A (2020). Comparative lipidomic study of human milk from different lactation stages and milk formulas. Nutrients.

[13] Calvo-Lerma J (2022). Breast milk lipidome is associated with maternal diet and infants' growth. Front. Nutr..

[14] Sinanoglou VJ (2017). Factors affecting human colostrum fatty acid profile: A case study. PLoS One.

[15] Marangoni F (2002). Polyunsaturated fatty acids in maternal plasma and in breast milk. Prostaglandins Leukot. Essent. Fatty Acids.

[16] Huang HL, Chuang LT, Li HH, Lin CP, Glew RH (2013). Docosahexaenoic acid in maternal and neonatal plasma phospholipids and milk lipids of Taiwanese women in Kinmen: Fatty acid composition of maternal blood, neonatal blood and breast milk. Lipids Health Dis..

[17] Meneses F, Torres AG, Trugo NM (2008). Essential and long-chain polyunsaturated fatty acid status and fatty acid composition of breast milk of lactating adolescents. Br. J. Nutr..

[18] LaBarre JL (2020). Maternal lipid levels across pregnancy impact the umbilical cord blood lipidome and infant birth weight. Sci. Rep..

[19] Bode L, Raman AS, Murch SH, Rollins NC, Gordon JI (2020). Understanding the mother-breastmilk-infant “triad”. Science.

[20] Christian P (2021). The need to study human milk as a biological system. Am. J. Clin. Nutr..

[21] Jensen CL, Maude M, Anderson RE, Heird WC (2000). Effect of docosahexaenoic acid supplementation of lactating women on the fatty acid composition of breast milk lipids and maternal and infant plasma phospholipids. Am. J. Clin. Nutr..

[22] Furse S (2019). Relationship between the lipid composition of maternal plasma and infant plasma through breast milk. Metabolomics.

[23] Hachey DL (1987). Human lactation: Maternal transfer of dietary triglycerides labeled with stable isotopes. J. Lipid Res..

[24] Leat WM, Kubasek FO, Buttress N (1976). Plasma lipoproteins of lambs and sheep. Q. J. Exp. Physiol. Cogn. Med. Sci..

[25] Ma X (2022). Phosphatidylserine, inflammation, and central nervous system diseases. Front. Aging Neurosci..

[26] Brezinova M (2018). Levels of palmitic acid ester of hydroxystearic acid (PAHSA) are reduced in the breast milk of obese mothers. Biochim. Biophys. Acta Mol. Cell Biol. Lipids.

[27] Yore MM (2014). Discovery of a class of endogenous mammalian lipids with anti-diabetic and anti-inflammatory effects. Cell.

[28] Cavender CP, Turley SD, Dietschy JM (1995). Sterol metabolism in fetal, newborn, and suckled lambs and their response to cholesterol after weaning. Am. J. Physiol..

[29] Argov-Argaman N (2010). Lactosomes: Structural and compositional classification of unique nanometer-sized protein lipid particles of human milk. J. Agric. Food Chem..

[30] German JB, Argov-Argaman N, Boyd BJ (2019). Milk lipids: A complex nutrient delivery system. Nestle Nutr. Inst. Workshop Ser..

[31] Rudolph MC, McManaman JL, Hunter L, Phang T, Neville MC (2003). Functional development of the mammary gland: use of expression profiling and trajectory clustering to reveal changes in gene expression during pregnancy, lactation, and involution. J Mammary Gland. Biol. Neoplas..

[32] Neville MC, Picciano MF (1997). Regulation of milk lipid secretion and composition. Annu. Rev. Nutr..

[33] Lane MA, Baldwin RT, Jesse BW (2000). Sheep rumen metabolic development in response to age and dietary treatments. J. Anim. Sci..

[34] Di Gerlando R (2019). Genome-wide association study between CNVs and milk production traits in Valle del Belice sheep. PLoS One.

[35] Mu T (2022). Screening and conjoint analysis of key lncRNAs for milk fat metabolism in dairy cows. Front. Genet..

[36] van den Berg I (2020). Meta-analysis for milk fat and protein percentage using imputed sequence variant genotypes in 94,321 cattle from eight cattle breeds. Genet. Sel. Evol..

[37] Much D (2013). Breast milk fatty acid profile in relation to infant growth and body composition: results from the INFAT study. Pediatr. Res..

[38] Manninen S (2022). Carnitine intake and serum levels associate positively with postnatal growth and brain size at term in very preterm infants. Nutrients.

[39] Zhou H, Huo Y, Yang N, Wei T (2023). Phosphatidic acid: From biophysical properties to diverse functions. FEBS J..

[40] Liu Z (2016). Identification and quantification of phosphatidylinositol in infant formulas by liquid chromatography-mass spectrometry. Food Chem..

[41] Wolfs D (2021). Brown fat-activating lipokine 12,13-diHOME in human milk is associated with infant adiposity. J. Clin. Endocrinol. Metab..

[42] Pekmez CT (2020). Breastmilk lipids and oligosaccharides influence branched short-chain fatty acid concentrations in infants with excessive weight gain. Mol. Nutr. Food Res..

[43] Enstad S (2021). The impact of maternal obesity and breast milk inflammation on developmental programming of infant growth. Eur. J. Clin. Nutr..

[44] Vidakovic AJ (2017). Maternal plasma polyunsaturated fatty acid levels during pregnancy and childhood lipid and insulin levels. Nutr. Metab. Cardiovasc. Dis..

[45] Hellmuth C (2019). Maternal metabolomic profile and fetal programming of offspring adiposity: Identification of potentially protective lipid metabolites. Mol. Nutr. Food Res..

[46] Baldwin RL, McLeod KR, Klotz JL, Heitmann RN (2004). Rumen development, intestinal growth and hepatic metabolism in the pre- and postweaning ruminant. J. Dairy Sci..

[47] Zhao L, Zhang J, Ge W, Wang J (2022). Comparative lipidomics analysis of human and ruminant milk reveals variation in composition and structural characteristics. J. Agric. Food Chem..

[48] Bayril T (2023). Effects of lamb sex, parity, and birth type on milk yield, lactation length, and milk components in Zom ewes raised under semi-intensive conditions. S. Afr. J. Anim. Sci..

[49] Ventrella D (2021). Animal models for in vivo lactation studies: Anatomy, physiology and milk compositions in the most used non-clinical species—A contribution from the ConcePTION project. Animals.

[50] Laliotis GP, Bizelis I, Rogdakis E (2010). Comparative approach of the de novo fatty acid synthesis (lipogenesis) between ruminant and non ruminant mammalian species: From biochemical level to the main regulatory lipogenic genes. Curr. Genom..

[51] Liu ZQ, Rochfort S (2023). Lipidomics in milk: Recent advances and developments. Curr. Opin. Food Sci..

[52] Percie du Sert N (2020). The ARRIVE guidelines 2.0: Updated guidelines for reporting animal research. BMJ Open Sci..

[53] Manikkam M (2004). Fetal programming: prenatal testosterone excess leads to fetal growth retardation and postnatal catch-up growth in sheep. Endocrinology.

[54] Snowder GD, Glimp HA (1991). Influence of breed, number of suckling lambs, and stage of lactation on ewe milk production and lamb growth under range conditions. J. Anim. Sci/.

[55] Bligh EG, Dyer WJ (1959). A rapid method of total lipid extraction and purification. Can. J. Biochem. Physiol..

[56] Gika HG, Macpherson E, Theodoridis GA, Wilson ID (2008). Evaluation of the repeatability of ultra-performance liquid chromatography-TOF-MS for global metabolic profiling of human urine samples. J. Chromatogr. B Analyt. Technol. Biomed. Life Sci..

[57] Chambers MC (2012). A cross-platform toolkit for mass spectrometry and proteomics. Nat. Biotechnol..

[58] Kind T (2012). Qualitative analysis of algal secretions with multiple mass spectrometric platforms. J. Chromatogr. A.

[59] Meissen JK (2012). Induced pluripotent stem cells show metabolomic differences to embryonic stem cells in polyunsaturated phosphatidylcholines and primary metabolism. PLoS One.

[60] Ejsing CS (2006). Automated identification and quantification of glycerophospholipid molecular species by multiple precursor ion scanning. Anal. Chem..

[61] Pang Z (2021). MetaboAnalyst 5.0: Narrowing the gap between raw spectra and functional insights. Nucleic Acids Res..

[62] Baek J, He C, Afshinnia F, Michailidis G, Pennathur S (2022). Lipidomic approaches to dissect dysregulated lipid metabolism in kidney disease. Nat. Rev. Nephrol..

[63] Gaud C (2021). BioPAN: A web-based tool to explore mammalian lipidome metabolic pathways on LIPID MAPS. F1000Res.

