# Supplementary material for: Comparative lipidome study of maternal plasma, milk, and lamb plasma in sheep
Source: Sci Rep. 2024 Mar 28;14:7401. doi: 10.1038/s41598-024-58116-5 (PMC10978966; doi:10.1038/s41598-024-58116-5)
Supplement: Supplementary file 1 — Supplementary Information. [file 41598_2024_58116_MOESM1_ESM.docx]

**Supplementary Figures**

Supplementary Figure S1: **Multivariate analysis of** **maternal plasma, milk and lamb plasma lipidome.** A. OPLS-DA Score plot and B. VIP Score plot of lipidome comparison between maternal plasma and milk, C. OPLS-DA Score plot and D. VIP Score plot of lipidome comparison between lamb plasma and milk, E. OPLS-DA Score plot and F. VIP Score plot of lipidome comparison between maternal plasma and lamb plasma.


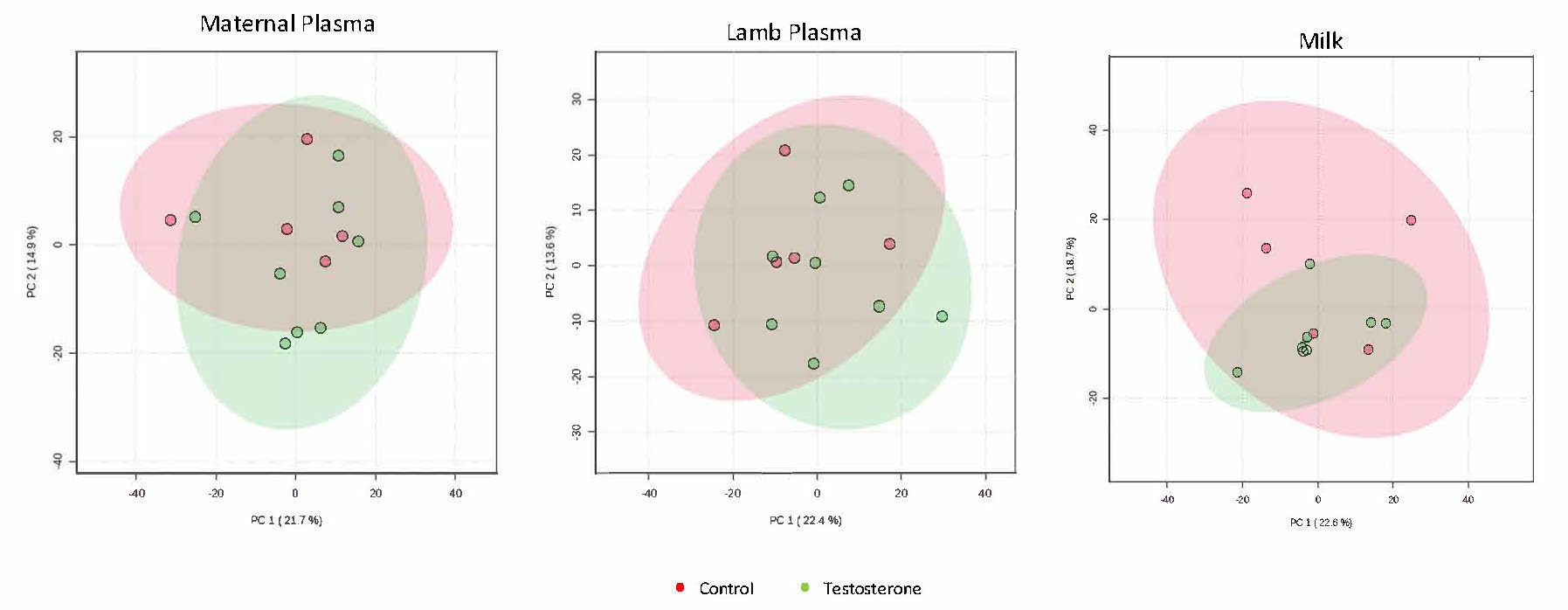


Supplementary Figure S2: **Effect of prenatal-T treatment on lipidome.** Principal Component Analysis score plots based on lipids compared between control (n=5) and prenatal-T (n=8) groups in maternal plasma, lamb plasma and milk.


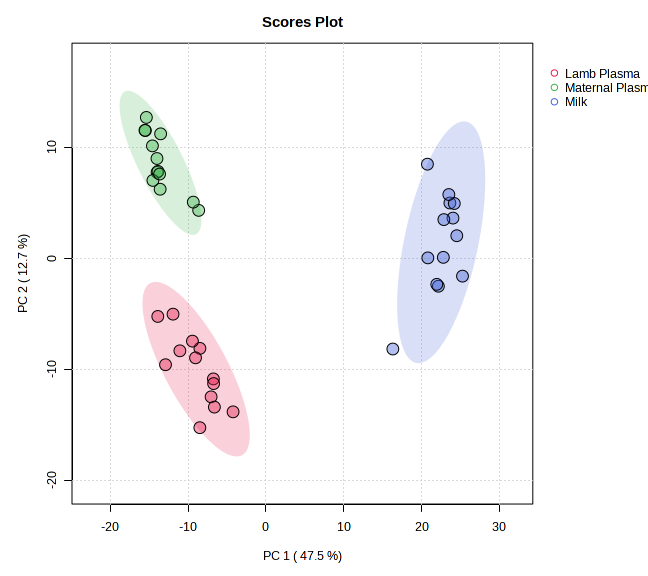


Supplementary Figure S3: **Principal Component Analysis plot showing comparison of lipids** **between milk, maternal plasma and lamb plasma.**

**Supplementary Table Legend:**

S1. Lipid Classes present in maternal plasma, lamb plasma and milk.

S2. Lipids found exclusively in milk compared to plasma.

S3. Lipids found exclusively in plasma compared to milk.

S4. Differential lipids in milk compared to maternal plasma.

S5. Differential lipids in milk compared to lamb plasma.

S6. Differential lipids in maternal plasma compared to lamb plasma.

S7. Correlated lipids between maternal plasma, milk and lamb plasma.

S8. Active and suppressed pathways based on lipid class.

Supplementary Table S1. Lipid Classes present in maternal and lamb plasma and milk.

| **Lipid Classes in Plasma** | **Lipid Classes in Milk** | **Lipid Classes in Plasma and Milk** |
| --- | --- | --- |
| DG O- | CoQ | CAR |
| MG | Hex3Cer | CE |
| PEtOH | PI O- | Cer |
| PMeOH | MGDG | CerP |
| 25-hydroxycholecalciferol | MGDG O- | Cholesterol |
|  |  | DG |
|  |  | FAHFA |
|  |  | FFA |
|  |  | Hex2Cer |
|  |  | HexCer |
|  |  | LNAPE |
|  |  | LPA |
|  |  | LPC |
|  |  | LPE |
|  |  | LPC O- |
|  |  | LPE O- |
|  |  | PA |
|  |  | PC |
|  |  | PE |
|  |  | PI |
|  |  | PG |
|  |  | PS |
|  |  | PC O- |
|  |  | PE O- |
|  |  | PE-Cer |
|  |  | PE-NMe |
|  |  | PE-NMe2 |
|  |  | PG O- |
|  |  | SM |
|  |  | ST |
|  |  | TG |
|  |  | TG O- |

S2. Lipids found exclusively in milk compared to plasma

| CE 18:3 |
| --- |
| Cer 26:0;2O |
| Cer 26:1;2O |
| Cer 28:1;2O |
| Cer 30:1;2O |
| Cer 31:1;2O |
| Cer 32:0;2O |
| Cer 35:1;2O |
| Cer 35:2;2O |
| Cer 44:1;2O |
| CerP 38:1;2O |
| CerP 42:2;2O |
| CoQ10 |
| DG 17:0 |
| DG 18:0 |
| DG 19:0 |
| DG 20:0 |
| DG 21:0 |
| DG 22:0 |
| DG 23:0 |
| DG 24:1 |
| DG 25:0 |
| DG 26:2 |
| DG 26:3 |
| DG 26:4 |
| DG 28:2 |
| DG 28:3 |
| DG 28:4 |
| DG 30:2 |
| DG 30:3 |
| DG 30:5 |
| DG 32:2 |
| DG 32:3 |
| DG 32:5 |
| DG 32:6 |
| DG 34:5 |
| FAHFA 25:0 |
| FAHFA 26:0 |
| FAHFA 26:1 |
| FAHFA 27:0 |
| FAHFA 28:1 |
| FAHFA 29:0 |
| FAHFA 30:0 |
| FAHFA 30:1 |
| FAHFA 31:0 |
| FAHFA 31:1 |
| FAHFA 32:1 |
| FAHFA 33:1 |
| FAHFA 34:1 |
| FAHFA 34:2 |
| FAHFA 34:3 |
| FAHFA 36:2 |
| FAHFA 38:3 |
| FAHFA 44:3 |
| Hex2Cer 43:1;2O |
| Hex2Cer 43:2;2O |
| Hex2Cer 44:1;2O |
| Hex3Cer 34:1;2O |
| Hex3Cer 41:1;2O |
| HexCer 32:1;2O |
| HexCer 33:1;2O |
| HexCer 34:1;2O |
| HexCer 36:1;2O |
| HexCer 38:1;2O |
| HexCer 39:1;2O |
| HexCer 40:1;2O |
| HexCer 41:0;2O |
| HexCer 41:1;2O |
| HexCer 42:0;2O |
| HexCer 42:1;2O |
| HexCer 42:2;2O |
| HexCer 43:1;2O |
| LNAPE N-33:0 |
| LPC 17:0 |
| LPC 28:0 |
| LPC O-18:2 |
| LPC O-26:1 |
| LPE 20:2 |
| LPE 22:4 |
| LPE 34:1 |
| LPE 40:7 |
| MGDG 18:0 |
| MGDG O-28:0 |
| MGDG O-30:0 |
| MGDG O-31:1 |
| MGDG O-33:1 |
| PA 30:0 |
| PA 32:0 |
| PA 36:4 |
| PC 22:0 |
| PC 25:0 |
| PC 26:1 |
| PC 28:2 |
| PC 35:7 |
| PC 39:0 |
| PC 40:0 |
| PC 42:0 |
| PC 42:1 |
| PC 42:7 |
| PC 44:1 |
| PC O-12:0 |
| PC O-18:0 |
| PC O-18:1 |
| PC O-22:5 |
| PC O-33:0 |
| PC O-35:6 |
| PC O-36:0 |
| PC O-37:4 |
| PC O-38:0 |
| PC O-39:8 |
| PC O-40:0 |
| PC O-42:3 |
| PE 22:0 |
| PE 24:0 |
| PE 26:0 |
| PE 28:0 |
| PE 28:1 |
| PE 28:2 |
| PE 30:1 |
| PE 30:2 |
| PE 32:3 |
| PE 34:5 |
| PE 35:0 |
| PE 36:6 |
| PE 37:1 |
| PE 37:2 |
| PE 37:3 |
| PE 37:5 |
| PE 38:0 |
| PE 38:1 |
| PE 38:2 |
| PE 39:1 |
| PE 39:4 |
| PE 40:0 |
| PE 40:1 |
| PE 40:2 |
| PE 40:4 |
| PE 41:1 |
| PE 42:7 |
| PE O-26:1 |
| PE O-30:2 |
| PE O-36:0 |
| PE O-37:1 |
| PE O-37:4 |
| PE O-38:1 |
| PE O-40:1 |
| PE O-41:4 |
| PE O-41:6 |
| PE O-44:4 |
| PE O-44:7 |
| PE O-46:5 |
| PE-Cer 42:1;2O |
| PE-NMe 36:2 |
| PE-NMe 36:4 |
| PE-NMe 38:4 |
| PE-NMe2 34:4 |
| PE-NMe2 36:5 |
| PE-NMe2 38:4 |
| PG 32:1 |
| PG 34:0 |
| PG 34:1 |
| PG 38:1 |
| PG 38:2 |
| PG 38:3 |
| PG 38:5 |
| PG 40:3 |
| PG 40:4 |
| PG 40:5 |
| PG 42:8 |
| PG O-32:2 |
| PG O-36:0 |
| PI 35:1 |
| PI 36:0 |
| PI 37:2 |
| PI O-36:2 |
| PS 36:1 |
| PS 36:2 |
| PS 36:3 |
| PS 37:1 |
| PS 38:3 |
| PS 40:2 |
| PS 40:4 |
| PS 40:5 |
| PS 40:6 |
| PS 40:7 |
| SM 22:1;2O |
| SM 36:0;2O |
| SM 36:2;3O |
| SM 38:0;2O |
| SM 38:3;2O |
| SM 39:3;3O |
| SM 40:0;2O |
| SM 40:0;3O |
| SM 42:6;2O |
| SM 43:4;2O |
| SM 45:3;2O |
| ST 28:1;O;Hex;FA 16:0 |
| ST 28:1;O;Hex;FA 18:1 |
| TG 25:0 |
| TG 27:0 |
| TG 29:0 |
| TG 31:0 |
| TG 31:1 |
| TG 32:1 |
| TG 34:2 |
| TG 34:3 |
| TG 35:2 |
| TG 36:3 |
| TG 36:4 |
| TG 38:5 |
| TG 41:3 |
| TG 45:4 |
| TG 58:0 |
| TG 61:3 |
| TG O-34:1 |
| TG O-36:0 |
| TG O-36:1 |
|  |
|  |
|  |

S3. Lipids found exclusively in plasma compared to milk

| 25-hydroxy cholecalciferol |
| --- |
| CAR 12:1 |
| CAR 14:2 |
| CAR 17:0 |
| CAR 24:0 |
| CE 15:0 |
| CE 18:0 |
| CE 19:1 |
| CE 22:6 |
| Cer 35:4;2O |
| Cer 38:0;3O |
| Cer 39:0;2O |
| Cer 40:0;3O |
| CerP 34:0;2O |
| DG 33:0 |
| DG 33:1 |
| DG 35:0 |
| DG 35:1 |
| DG 38:1 |
| DG 38:3 |
| DG 38:4 |
| Hex2Cer 34:2;2O |
| HexCer 34:0;3O |
| HexCer 34:1;2O |
| HexCer 34:2;2O |
| HexCer 36:1;2O |
| HexCer 42:0;2O |
| LNAPE N-31:1 |
| LNAPE N-35:1 |
| LNAPE N-36:4 |
| LNAPE N-37:1 |
| LNAPE N-37:4 |
| LNAPE N-38:0 |
| LNAPE N-39:1 |
| LNAPE N-39:2 |
| LNAPE N-39:4 |
| LNAPE N-40:0 |
| LNAPE N-40:1 |
| LNAPE N-40:2 |
| LNAPE N-40:4 |
| LPA 17:0 |
| LPC 12:0 |
| LPC 16:2 |
| LPC 17:1 |
| LPC 18:4 |
| LPC 20:1 |
| LPC 21:0 |
| LPC 22:1 |
| LPC 22:2 |
| LPC 22:3 |
| LPC 23:0 |
| LPC 24:1 |
| LPC 24:5 |
| LPC 24:6 |
| LPC 26:0 |
| LPC 26:1 |
| LPC 26:2 |
| LPC 34:2 |
| LPC 36:2 |
| LPC 42:5 |
| LPC 42:6 |
| LPC O-14:1 |
| LPC O-17:0 |
| LPC O-17:2 |
| LPC O-20:1 |
| LPC O-22:0 |
| LPC O-22:1 |
| LPE 16:1 |
| LPE 20:1 |
| LPE 24:0 |
| LPE 34:2 |
| LPE 36:2 |
| LPE 37:0 |
| LPE 38:4 |
| LPE 38:5 |
| LPE O-20:1 |
| LPE O-22:1 |
| LPE O-24:2 |
| MG 26:4 |
| PA 36:1 |
| PA 36:2 |
| PA 36:5 |
| PA 38:4 |
| PC 26:3 |
| PC 28:3 |
| PC 29:1 |
| PC 31:2 |
| PC 32:3 |
| PC 32:4 |
| PC 33:3 |
| PC 33:4 |
| PC 33:5 |
| PC 37:1 |
| PC 37:3 |
| PC 37:7 |
| PC 38:0 |
| PC 38:1 |
| PC 38:9 |
| PC 39:3 |
| PC 39:7 |
| PC 40:10 |
| PC 40:3 |
| PC 41:1 |
| PC 41:2 |
| PC 41:4 |
| PC 41:5 |
| PC 41:7 |
| PC 42:10 |
| PC 42:3 |
| PC 42:4 |
| PC 42:5 |
| PC 42:6 |
| PC 44:10 |
| PC 44:11 |
| PC 44:2 |
| PC 44:4 |
| PC 44:5 |
| PC 44:6 |
| PC O-19:3 |
| PC O-20:0 |
| PC O-21:3 |
| PC O-22:0 |
| PC O-23:1 |
| PC O-28:3 |
| PC O-29:1 |
| PC O-30:3 |
| PC O-31:1 |
| PC O-32:1 |
| PC O-32:2 |
| PC O-32:3 |
| PC O-33:2 |
| PC O-33:3 |
| PC O-34:4 |
| PC O-34:6 |
| PC O-35:5 |
| PC O-36:6 |
| PC O-36:7 |
| PC O-36:9 |
| PC O-37:1 |
| PC O-37:6 |
| PC O-38:10 |
| PC O-38:6 |
| PC O-38:8 |
| PC O-39:4 |
| PC O-39:5 |
| PC O-39:6 |
| PC O-40:9 |
| PC O-42:2 |
| PC O-42:9 |
| PC O-43:4 |
| PC O-44:4 |
| PC O-46:7 |
| PE 33:0 |
| PE 37:4 |
| PE O-16:0 |
| PE O-18:1 |
| PE O-29:1 |
| PE O-34:6 |
| PE O-35:6 |
| PE O-44:6 |
| PE-Cer 39:1;2O |
| PE-NMe2 16:1 |
| PE-NMe2 32:1 |
| PEtOH 36:2 |
| PEtOH 36:3 |
| PEtOH 36:4 |
| PG 34:2 |
| PI 40:6 |
| PMeOH 34:1 |
| PMeOH 34:2 |
| SM 26:1;2O |
| SM 27:1;2O |
| SM 28:0;2O |
| SM 29:1;2O |
| SM 30:3;2O |
| SM 31:0;2O |
| SM 31:3;2O |
| SM 31:4;2O |
| SM 32:1;3O |
| SM 32:3;2O |
| SM 32:4;2O |
| SM 33:0;3O |
| SM 33:1;3O |
| SM 33:4;2O |
| SM 35:1;2O |
| SM 35:2;3O |
| SM 35:3;2O |
| SM 35:3;3O |
| SM 36:0;3O |
| SM 36:1;3O |
| SM 37:0;2O |
| SM 37:4;2O |
| SM 38:7;3O |
| SM 39:1;3O |
| SM 40:1;2O(FA 16:0) |
| SM 41:1;2O |
| SM 41:1;3O |
| SM 41:2;2O |
| SM 41:4;2O |
| SM 41:1;2O |
| SM 41:1;3O |
| SM 41:2;2O |
| SM 41:2;3O |
| SM 41:3;2O |
| SM 41:6;2O |
| SM 42:8;3O |
| SM 43:0;3O |
| SM 43:1;3O |
| SM 43:5;2O |
| SM 44:5;2O |
| SPB 18:1;2O |
| ST 24:1;O3;G |
| ST 24:1;O5 |
| ST 27:1;O;Hex |
| TG 42:4 |
| TG 42:5 |
| TG 46:6 |
| TG 47:3 |
| TG 49:5 |
| TG 51:0 |
| TG 52:7 |
| TG 53:6 |
| TG 54:0 |
| TG 54:9 |
| TG 55:0 |
| TG 55:1 |
| TG 55:4 |
| TG 55:5 |
| TG 56:9 |
| TG 57:2 |
| TG 57:5 |
| TG 57:7 |
| TG 57:9 |
| TG 58:10 |
| TG 59:1 |
| TG 59:2 |
| TG 59:4 |
| TG 60:10 |
| TG 60:12 |
| TG 60:8 |
| TG 60:9 |
| TG 62:1 |
| TG 63:5 |
| TG O-32:0 |
| TG O-34:0 |
|  |
|  |
|  |
|  |

S4. Differential lipids in milk compared to maternal plasma

| **Lipid Name** | **FC** | **log2(FC)** | **Adjusted P** |
| --- | --- | --- | --- |
| TG 24:0 | 13418.00 | 13.71 | 1.29E-17 |
| TG 40:0 | 2800.20 | 11.45 | 6.08E-15 |
| TG 36:0 | 3859.10 | 11.91 | 2.83E-13 |
| TG 40:1 | 8207.00 | 13.00 | 4.01E-13 |
| TG 38:0 | 1855.60 | 10.86 | 1.51E-12 |
| TG 36:2 | 10507.00 | 13.36 | 1.51E-12 |
| TG 35:1 | 2035.60 | 10.99 | 2.76E-12 |
| TG 42:1 | 2947.10 | 11.53 | 2.83E-12 |
| Cer 34:1;2O | 310.82 | 8.28 | 3.39E-12 |
| TG 44:3 | 1194.80 | 10.22 | 3.39E-12 |
| TG 34:1 | 20074.00 | 14.29 | 4.94E-12 |
| TG 42:2 | 2660.40 | 11.38 | 5.11E-12 |
| TG 41:1 | 1326.00 | 10.37 | 7.03E-12 |
| TG 48:2 | 87.32 | 6.45 | 7.81E-12 |
| TG 39:1 | 2698.70 | 11.40 | 1.01E-11 |
| TG 44:2 | 1702.00 | 10.73 | 1.33E-11 |
| Hex2Cer 41:1;2O | 78.07 | 6.29 | 1.35E-11 |
| TG 42:0 | 623.52 | 9.28 | 1.79E-11 |
| TG 42:3 | 2937.20 | 11.52 | 1.79E-11 |
| TG 45:1 | 94.01 | 6.55 | 1.82E-11 |
| PE O-36:2 | 141.30 | 7.14 | 2.47E-11 |
| PI 37:4 | 59.50 | 5.89 | 2.47E-11 |
| TG 41:0 | 393.23 | 8.62 | 4.00E-11 |
| TG 38:1 | 7155.20 | 12.81 | 4.24E-11 |
| PC 38:4 | 0.02 | -5.91 | 4.43E-11 |
| TG 38:3 | 3925.70 | 11.94 | 4.43E-11 |
| TG 35:0 | 5265.80 | 12.36 | 5.70E-11 |
| TG 44:1 | 1462.10 | 10.51 | 6.34E-11 |
| PC 36:2 | 0.11 | -3.16 | 7.49E-11 |
| TG 37:0 | 2828.10 | 11.47 | 9.85E-11 |
| LPC 22:5 | 0.02 | -5.66 | 1.45E-10 |
| TG 43:2 | 171.23 | 7.42 | 1.61E-10 |
| LNAPE N-36:0 | 9.29 | 3.22 | 1.61E-10 |
| TG 43:3 | 82.75 | 6.37 | 1.91E-10 |
| PC 38:5 | 0.06 | -4.01 | 1.97E-10 |
| PC 36:5 | 0.02 | -5.41 | 2.28E-10 |
| TG 43:1 | 1113.40 | 10.12 | 2.38E-10 |
| TG 46:1 | 105.29 | 6.72 | 2.92E-10 |
| TG 33:0 | 6777.60 | 12.73 | 3.16E-10 |
| PC 36:1 | 0.17 | -2.59 | 3.27E-10 |
| PC 40:5 | 0.05 | -4.39 | 3.99E-10 |
| LPC 18:0 | 0.04 | -4.75 | 4.32E-10 |
| TG 37:1 | 2892.40 | 11.50 | 4.81E-10 |
| TG 40:3 | 5388.70 | 12.40 | 5.02E-10 |
| Cer 35:0;2O | 0.16 | -2.63 | 5.29E-10 |
| DG 32:1 | 35.44 | 5.15 | 6.17E-10 |
| PE O-40:6 | 10.07 | 3.33 | 6.88E-10 |
| PE 40:6 | 55.64 | 5.80 | 7.45E-10 |
| PE 34:1 | 26.04 | 4.70 | 8.05E-10 |
| DG 30:1 | 278.92 | 8.12 | 9.53E-10 |
| DG 24:0 | 670.18 | 9.39 | 9.96E-10 |
| TG 44:4 | 429.86 | 8.75 | 1.19E-09 |
| PC 38:3 | 0.04 | -4.48 | 1.30E-09 |
| TG 45:2 | 87.82 | 6.46 | 1.32E-09 |
| PC 40:6 | 0.11 | -3.19 | 1.33E-09 |
| TG 48:5 | 57.14 | 5.84 | 1.38E-09 |
| PE 35:1 | 66.62 | 6.06 | 1.43E-09 |
| TG 39:0 | 2265.40 | 11.15 | 1.61E-09 |
| DG 26:0 | 419.23 | 8.71 | 1.81E-09 |
| TG 41:2 | 371.04 | 8.54 | 1.93E-09 |
| TG 48:1 | 18.89 | 4.24 | 2.23E-09 |
| PC 37:2 | 0.11 | -3.19 | 2.46E-09 |
| PC O-36:5 | 0.02 | -5.59 | 2.96E-09 |
| TG 50:6 | 19.46 | 4.28 | 3.23E-09 |
| DG 38:5 | 24.74 | 4.63 | 3.51E-09 |
| PE 36:3 | 7.42 | 2.89 | 3.57E-09 |
| DG 36:5 | 36.30 | 5.18 | 3.82E-09 |
| PE 36:4 | 48.61 | 5.60 | 4.30E-09 |
| LPC 16:0 | 0.09 | -3.46 | 4.46E-09 |
| PC 34:2 | 73.53 | 6.20 | 5.37E-09 |
| TG 46:3 | 259.95 | 8.02 | 5.49E-09 |
| TG 40:2 | 30570.00 | 14.90 | 6.41E-09 |
| PE 36:1 | 23.61 | 4.56 | 6.58E-09 |
| DG 28:1 | 592.91 | 9.21 | 8.37E-09 |
| PI 36:1 | 5.56 | 2.48 | 8.53E-09 |
| TG 43:0 | 111.57 | 6.80 | 9.24E-09 |
| PE 32:1 | 73.62 | 6.20 | 1.01E-08 |
| TG 46:0 | 108.95 | 6.77 | 1.22E-08 |
| DG 28:0 | 218.39 | 7.77 | 1.28E-08 |
| PE O-34:2 | 9.47 | 3.24 | 1.28E-08 |
| TG 48:6 | 81.08 | 6.34 | 1.28E-08 |
| PE 40:8 | 10.52 | 3.40 | 1.41E-08 |
| TG 36:1 | 22811.00 | 14.48 | 1.49E-08 |
| PC 34:3 | 0.06 | -3.96 | 1.56E-08 |
| TG 38:2 | 23470.00 | 14.52 | 1.58E-08 |
| TG 47:2 | 18.26 | 4.19 | 1.69E-08 |
| LPC O-16:1 | 0.08 | -3.70 | 1.71E-08 |
| Cer 41:2;2O | 43.54 | 5.44 | 1.99E-08 |
| PE 30:0 | 58.46 | 5.87 | 1.99E-08 |
| PE O-37:7 | 0.05 | -4.31 | 1.99E-08 |
| TG 48:3 | 34.51 | 5.11 | 1.99E-08 |
| TG 46:5 | 69.25 | 6.11 | 2.02E-08 |
| TG 51:3 | 382.47 | 8.58 | 2.09E-08 |
| PC 33:0 | 6.31 | 2.66 | 2.76E-08 |
| Hex2Cer 34:1;2O | 8.97 | 3.16 | 2.83E-08 |
| LPC O-16:0 | 0.05 | -4.32 | 3.06E-08 |
| PE O-34:3 | 9.55 | 3.26 | 3.15E-08 |
| SM 38:1;2O | 4.32 | 2.11 | 3.15E-08 |
| DG 26:1 | 203.28 | 7.67 | 3.40E-08 |
| LNAPE N-36:1 | 0.05 | -4.46 | 3.40E-08 |
| DG 34:3 | 23.56 | 4.56 | 3.79E-08 |
| PE 38:4 | 10.25 | 3.36 | 3.79E-08 |
| DG 36:2 | 9.56 | 3.26 | 3.97E-08 |
| PE 35:2 | 8.09 | 3.02 | 3.97E-08 |
| PC 38:2 | 0.20 | -2.35 | 4.17E-08 |
| SM 39:1;2O | 8.62 | 3.11 | 4.47E-08 |
| TG 50:5 | 10.95 | 3.45 | 5.06E-08 |
| PE 32:0 | 14.33 | 3.84 | 5.68E-08 |
| TG 54:4 | 6.85 | 2.78 | 5.76E-08 |
| LPC 24:0 | 0.05 | -4.46 | 6.00E-08 |
| TG 48:4 | 27.41 | 4.78 | 6.90E-08 |
| SM 34:2;2O | 0.11 | -3.16 | 7.31E-08 |
| LPC 20:3 | 0.01 | -7.09 | 9.30E-08 |
| PC 36:4 | 0.07 | -3.81 | 1.09E-07 |
| PE O-35:2 | 10.64 | 3.41 | 1.10E-07 |
| LPC 22:4 | 0.00 | -8.05 | 1.13E-07 |
| Cer 39:1;2O | 6.54 | 2.71 | 1.39E-07 |
| TG 50:2 | 10.53 | 3.40 | 1.43E-07 |
| LPC 15:0 | 0.03 | -5.01 | 1.88E-07 |
| PE O-37:2 | 13.71 | 3.78 | 2.06E-07 |
| SM 34:0;2O | 5.41 | 2.44 | 2.07E-07 |
| LNAPE N-36:2 | 0.01 | -6.42 | 2.25E-07 |
| TG 50:4 | 12.35 | 3.63 | 2.25E-07 |
| PC O-34:1 | 0.14 | -2.83 | 2.62E-07 |
| PE O-36:1 | 6.40 | 2.68 | 2.95E-07 |
| DG 30:0 | 81.05 | 6.34 | 3.21E-07 |
| PC 35:4 | 0.02 | -5.71 | 3.21E-07 |
| PC O-36:4 | 0.08 | -3.68 | 3.21E-07 |
| PE O-36:3 | 6.44 | 2.69 | 3.50E-07 |
| PC 35:5 | 0.04 | -4.59 | 3.51E-07 |
| PE O-38:8 | 0.11 | -3.23 | 3.51E-07 |
| DG 36:4 | 24.42 | 4.61 | 3.83E-07 |
| PC O-38:5 | 0.04 | -4.62 | 3.83E-07 |
| LPC 19:0 | 0.16 | -2.63 | 3.87E-07 |
| PE-Cer 42:3;2O | 10.09 | 3.33 | 4.51E-07 |
| PC 32:0 | 5.18 | 2.37 | 4.71E-07 |
| LPC O-17:1 | 0.13 | -2.89 | 4.89E-07 |
| LPC 18:1 | 0.10 | -3.36 | 5.19E-07 |
| LPC O-18:1 | 0.05 | -4.33 | 5.20E-07 |
| LPC 18:3 | 0.05 | -4.45 | 6.13E-07 |
| LNAPE N-37:2 | 0.01 | -7.36 | 6.38E-07 |
| LPC 20:5 | 0.01 | -6.73 | 6.51E-07 |
| TG 47:1 | 18.32 | 4.19 | 6.70E-07 |
| PC 37:4 | 0.06 | -4.10 | 6.74E-07 |
| PE 36:2 | 4.91 | 2.30 | 6.74E-07 |
| PC 40:4 | 0.20 | -2.35 | 7.51E-07 |
| TG 52:2 | 7.44 | 2.90 | 7.83E-07 |
| PE O-38:2 | 3.16 | 1.66 | 7.94E-07 |
| TG 53:2 | 8.37 | 3.07 | 9.53E-07 |
| DG 36:3 | 10.92 | 3.45 | 9.72E-07 |
| LPC 18:2 | 0.04 | -4.83 | 9.87E-07 |
| TG 54:2 | 8.00 | 3.00 | 9.98E-07 |
| LPC 19:1 | 0.07 | -3.82 | 1.03E-06 |
| PC 29:0 | 14.64 | 3.87 | 1.03E-06 |
| Cer 41:0;3O | 0.07 | -3.75 | 1.08E-06 |
| LPC 22:6 | 0.08 | -3.56 | 1.08E-06 |
| DG 38:0 | 0.05 | -4.45 | 1.14E-06 |
| Hex2Cer 42:1;2O | 0.20 | -2.33 | 1.14E-06 |
| TG 45:3 | 22.04 | 4.46 | 1.15E-06 |
| TG 46:4 | 14.72 | 3.88 | 1.32E-06 |
| PE O-40:8 | 5.71 | 2.51 | 1.52E-06 |
| PC O-40:8 | 0.08 | -3.59 | 1.55E-06 |
| PC 32:2 | 0.09 | -3.54 | 1.61E-06 |
| LPC 20:4 | 0.02 | -5.82 | 1.69E-06 |
| DG 32:0 | 16.42 | 4.04 | 1.84E-06 |
| LPC O-18:0 | 0.01 | -6.37 | 1.88E-06 |
| SM 32:0;2O | 6.22 | 2.64 | 2.02E-06 |
| PC 34:5 | 0.14 | -2.88 | 2.14E-06 |
| LPC 16:1 | 0.11 | -3.16 | 2.51E-06 |
| TG 45:0 | 19.48 | 4.28 | 2.82E-06 |
| PC O-40:7 | 0.13 | -2.94 | 2.88E-06 |
| PI 34:2 | 5.57 | 2.48 | 2.90E-06 |
| PC O-34:2 | 0.14 | -2.80 | 2.99E-06 |
| TG 52:6 | 7.11 | 2.83 | 3.06E-06 |
| DG 34:4 | 9.25 | 3.21 | 3.33E-06 |
| PC O-36:3 | 0.17 | -2.59 | 3.33E-06 |
| PC O-38:4 | 0.07 | -3.78 | 3.40E-06 |
| PC O-37:5 | 0.07 | -3.79 | 3.60E-06 |
| PC 34:0 | 12.92 | 3.69 | 3.72E-06 |
| PC O-40:5 | 0.12 | -3.09 | 4.07E-06 |
| PE 31:1 | 9.91 | 3.31 | 4.16E-06 |
| PE O-32:2 | 3.48 | 1.80 | 4.16E-06 |
| PE-Cer 34:0;2O | 0.06 | -3.98 | 4.39E-06 |
| TG 46:2 | 9.63 | 3.27 | 4.60E-06 |
| PI 36:2 | 3.37 | 1.75 | 4.94E-06 |
| CAR 18:0 | 0.23 | -2.11 | 5.19E-06 |
| PC 37:5 | 0.21 | -2.25 | 5.33E-06 |
| SM 30:1;2O | 3.46 | 1.79 | 5.47E-06 |
| TG 60:1 | 92.58 | 6.53 | 6.18E-06 |
| TG 54:6 | 4.90 | 2.29 | 6.25E-06 |
| LNAPE N-32:0 | 6.36 | 2.67 | 6.65E-06 |
| DG 34:2 | 110.27 | 6.79 | 6.80E-06 |
| TG 60:3 | 8.22 | 3.04 | 6.80E-06 |
| TG 49:2 | 5.17 | 2.37 | 7.17E-06 |
| DG 38:6 | 28.41 | 4.83 | 8.22E-06 |
| PE 40:7 | 7.11 | 2.83 | 9.02E-06 |
| PE O-40:2 | 18.26 | 4.19 | 9.35E-06 |
| PE-NMe 35:4 | 133.52 | 7.06 | 9.58E-06 |
| TG 53:1 | 4.64 | 2.21 | 9.81E-06 |
| LNAPE N-37:3 | 0.06 | -4.14 | 1.21E-05 |
| PC 30:0 | 4.70 | 2.23 | 1.28E-05 |
| PE-Cer 36:1;2O | 0.17 | -2.58 | 1.29E-05 |
| TG 49:1 | 5.39 | 2.43 | 1.41E-05 |
| TG 58:1 | 21.86 | 4.45 | 1.49E-05 |
| PE 34:3 | 4.73 | 2.24 | 1.50E-05 |
| PI 34:1 | 3.90 | 1.96 | 1.60E-05 |
| PC O-39:7 | 0.11 | -3.22 | 1.88E-05 |
| LNAPE N-34:0 | 17.45 | 4.13 | 1.95E-05 |
| CerP 32:1;2O | 3.48 | 1.80 | 2.01E-05 |
| PS 38:4 | 8.30 | 3.05 | 2.17E-05 |
| PE O-35:4 | 2.71 | 1.44 | 2.19E-05 |
| PE O-34:1 | 4.62 | 2.21 | 2.22E-05 |
| LNAPE N-38:3 | 0.03 | -4.95 | 2.23E-05 |
| CAR 18:1 | 0.16 | -2.68 | 2.30E-05 |
| ST 27:1;O;S | 0.36 | -1.49 | 2.84E-05 |
| TG 51:2 | 5.76 | 2.53 | 2.95E-05 |
| PC O-40:6 | 0.15 | -2.72 | 2.97E-05 |
| TG 58:2 | 8.81 | 3.14 | 3.05E-05 |
| Cer 43:2;2O | 3.08 | 1.62 | 3.16E-05 |
| TG 61:2 | 19.35 | 4.27 | 3.23E-05 |
| TG 62:2 | 9.86 | 3.30 | 3.72E-05 |
| PE 38:6 | 3.59 | 1.84 | 3.76E-05 |
| TG 54:5 | 6.08 | 2.61 | 3.76E-05 |
| TG 56:2 | 4.25 | 2.09 | 3.79E-05 |
| LNAPE N-38:2 | 0.07 | -3.87 | 4.36E-05 |
| LPE 20:4 | 0.05 | -4.30 | 4.64E-05 |
| SM 28:1;2O | 3.90 | 1.96 | 4.64E-05 |
| PE O-38:4 | 3.06 | 1.61 | 5.63E-05 |
| Cer 38:2;2O | 3.45 | 1.79 | 5.76E-05 |
| TG 54:7 | 4.07 | 2.03 | 5.80E-05 |
| PG 33:0 | 0.21 | -2.25 | 6.08E-05 |
| PE 39:5 | 7.98 | 3.00 | 6.56E-05 |
| TG 26:0 | 10.44 | 3.38 | 6.67E-05 |
| Cer 36:2;2O | 2.95 | 1.56 | 6.95E-05 |
| LNAPE N-40:3 | 0.05 | -4.20 | 7.10E-05 |
| Cer 40:1;2O | 2.93 | 1.55 | 7.13E-05 |
| SM 34:1;3O | 3.38 | 1.76 | 7.47E-05 |
| TG 52:5 | 6.00 | 2.59 | 7.66E-05 |
| PE O-35:3 | 2.94 | 1.55 | 7.82E-05 |
| PC O-34:0 | 0.15 | -2.71 | 9.80E-05 |
| PE O-40:4 | 11.08 | 3.47 | 1.08E-04 |
| LNAPE N-35:2 | 0.13 | -2.91 | 1.09E-04 |
| TG 48:0 | 14.47 | 3.86 | 1.12E-04 |
| PC 39:5 | 0.12 | -3.01 | 1.16E-04 |
| TG 60:2 | 9.02 | 3.17 | 1.27E-04 |
| PE O-33:4 | 0.23 | -2.11 | 1.28E-04 |
| PC O-33:6 | 2.39 | 1.26 | 1.35E-04 |
| TG 55:3 | 3.60 | 1.85 | 1.35E-04 |
| Cer 34:0;3O | 0.18 | -2.45 | 1.36E-04 |
| TG 57:1 | 8.97 | 3.16 | 1.61E-04 |
| TG 56:7 | 4.48 | 2.16 | 1.66E-04 |
| PE O-31:1 | 0.43 | -1.20 | 1.96E-04 |
| PE 32:2 | 9.68 | 3.28 | 2.14E-04 |
| PE 40:3 | 0.21 | -2.28 | 2.15E-04 |
| PC 40:9 | 0.20 | -2.33 | 2.21E-04 |
| Cer 42:2;2O | 0.30 | -1.73 | 2.22E-04 |
| PC 31:0 | 0.21 | -2.23 | 2.25E-04 |
| PC 20:0 | 3.43 | 1.78 | 2.25E-04 |
| TG 47:0 | 3.05 | 1.61 | 2.30E-04 |
| PC O-35:3 | 0.19 | -2.43 | 2.36E-04 |
| PC 28:0 | 2.88 | 1.53 | 2.45E-04 |
| PC 39:1 | 2.83 | 1.50 | 2.60E-04 |
| SM 38:2;2O | 0.24 | -2.05 | 2.64E-04 |
| PC O-37:7 | 3.94 | 1.98 | 2.76E-04 |
| PE O-34:5 | 0.25 | -2.01 | 2.80E-04 |
| PC 33:2 | 0.13 | -2.91 | 2.85E-04 |
| PE O-18:0 | 2.53 | 1.34 | 2.85E-04 |
| PE O-42:9 | 3.30 | 1.72 | 2.85E-04 |
| PC O-36:2 | 0.25 | -1.98 | 2.97E-04 |
| TG 59:3 | 5.36 | 2.42 | 3.12E-04 |
| TG 53:3 | 5.29 | 2.40 | 3.22E-04 |
| PC O-42:6 | 0.19 | -2.42 | 3.35E-04 |
| PC 26:0 | 4.03 | 2.01 | 3.43E-04 |
| Cer 42:1;2O | 2.90 | 1.54 | 3.96E-04 |
| PE O-40:3 | 4.52 | 2.18 | 4.02E-04 |
| PE 38:3 | 5.80 | 2.54 | 4.09E-04 |
| TG 58:3 | 3.78 | 1.92 | 4.19E-04 |
| TG 56:1 | 4.69 | 2.23 | 4.20E-04 |
| PI 38:6 | 5.10 | 2.35 | 4.32E-04 |
| TG 60:4 | 4.42 | 2.14 | 4.39E-04 |
| PG 40:0 | 0.14 | -2.85 | 4.72E-04 |
| TG 52:0 | 8.39 | 3.07 | 5.22E-04 |
| TG 62:3 | 13.16 | 3.72 | 5.34E-04 |
| CAR 15:0 | 0.41 | -1.27 | 6.62E-04 |
| LNAPE N-36:3 | 0.05 | -4.32 | 7.56E-04 |
| PE O-34:4 | 0.39 | -1.34 | 8.19E-04 |
| LPE O-24:1 | 0.30 | -1.72 | 8.51E-04 |
| PE O-31:2 | 0.23 | -2.13 | 8.51E-04 |
| TG 50:0 | 0.15 | -2.75 | 8.67E-04 |
| PC 31:1 | 2.61 | 1.39 | 9.23E-04 |
| LPC 15:1 | 3.15 | 1.65 | 9.28E-04 |
| PC 27:0 | 2.90 | 1.53 | 9.66E-04 |
| PC O-35:1 | 0.21 | -2.27 | 1.09E-03 |
| PE 34:4 | 4.97 | 2.31 | 1.13E-03 |
| PE O-39:5 | 0.38 | -1.38 | 1.19E-03 |
| SM 34:3;2O | 0.10 | -3.27 | 1.22E-03 |
| Cer 34:2;2O | 2.48 | 1.31 | 1.23E-03 |
| Cer 36:0;2O | 4.56 | 2.19 | 1.23E-03 |
| TG 57:3 | 3.09 | 1.63 | 1.24E-03 |
| DG 36:1 | 11.68 | 3.55 | 1.30E-03 |
| TG 55:2 | 5.09 | 2.35 | 1.30E-03 |
| PE O-42:5 | 0.13 | -2.97 | 1.50E-03 |
| CAR 16:0 | 0.33 | -1.62 | 1.50E-03 |
| TG 60:0 | 32.80 | 5.04 | 1.54E-03 |
| LPA 18:0 | 0.21 | -2.26 | 1.58E-03 |
| Cer 32:1;2O | 2.20 | 1.14 | 1.60E-03 |
| LPC 20:2 | 0.22 | -2.21 | 1.61E-03 |
| TG 49:3 | 3.44 | 1.78 | 1.72E-03 |
| PE O-38:3 | 2.83 | 1.50 | 1.77E-03 |
| LPE 18:3 | 0.15 | -2.72 | 2.31E-03 |
| TG 56:8 | 2.92 | 1.55 | 2.49E-03 |
| PC 28:1 | 2.74 | 1.45 | 2.72E-03 |
| PE 31:0 | 3.37 | 1.75 | 2.73E-03 |
| PA 38:6 | 0.19 | -2.38 | 2.86E-03 |
| PC O-32:0 | 0.16 | -2.67 | 2.95E-03 |
| TG 52:1 | 8.19 | 3.03 | 3.24E-03 |
| PC 30:3 | 2.70 | 1.43 | 3.41E-03 |
| LPE 20:5 | 0.21 | -2.26 | 3.97E-03 |
| PC 36:6 | 0.22 | -2.15 | 4.50E-03 |
| PG 38:4 | 0.31 | -1.70 | 4.74E-03 |
| PE-Cer 32:1;2O | 3.35 | 1.74 | 4.83E-03 |
| Cer 34:0;2O | 2.19 | 1.13 | 4.85E-03 |
| TG 49:0 | 5.87 | 2.55 | 4.85E-03 |
| TG 54:8 | 5.73 | 2.52 | 5.44E-03 |
| SM 35:1;3O | 7.40 | 2.89 | 5.48E-03 |
| ST 24:1;O4 | 4.41 | 2.14 | 6.21E-03 |
| PE O-35:5 | 0.33 | -1.58 | 8.82E-03 |
| SM 36:4;2O | 0.19 | -2.41 | 1.05E-02 |
| LNAPE N-38:4 | 0.17 | -2.53 | 1.05E-02 |
| LPE 22:6 | 0.23 | -2.15 | 1.07E-02 |
| TG 61:1 | 8.52 | 3.09 | 1.09E-02 |
| TG 58:6 | 2.07 | 1.05 | 1.12E-02 |
| CAR 14:1 | 0.39 | -1.36 | 1.33E-02 |
| PC O-34:3 | 0.17 | -2.60 | 1.38E-02 |
| LPE 20:3 | 0.29 | -1.76 | 1.43E-02 |
| SM 37:1;2O | 5.39 | 2.43 | 1.53E-02 |
| FFA(24:2) | 0.37 | -1.45 | 1.57E-02 |
| TG 56:0 | 3.36 | 1.75 | 1.67E-02 |
| PC 24:0 | 3.15 | 1.65 | 2.08E-02 |
| LPC O-24:0 | 0.23 | -2.14 | 2.37E-02 |
| TG 51:5 | 2.28 | 1.19 | 2.49E-02 |
| PC 40:8 | 0.31 | -1.71 | 2.66E-02 |
| FFA(20:2) | 0.50 | -1.00 | 2.75E-02 |
| FFA(18:2) | 0.43 | -1.21 | 2.78E-02 |
| Cer 42:1;3O | 0.44 | -1.18 | 4.67E-02 |
| Cer 42:0;3O | 2.24 | 1.17 | 4.87E-02 |

S5. Differential lipids in milk compared to lamb plasma

| **Lipid Name** | **FC** | **log2(FC)** | **Adjusted P** |
| --- | --- | --- | --- |
| TG 24:0 | 18382.00 | 14.17 | 1.69E-10 |
| TG 40:3 | 479.58 | 8.91 | 1.69E-10 |
| PC 38:4 | 0.01 | -6.25 | 1.69E-10 |
| PE O-36:2 | 367.30 | 8.52 | 2.32E-10 |
| PC 36:2 | 0.09 | -3.55 | 3.35E-10 |
| TG 34:1 | 1730.50 | 10.76 | 3.39E-10 |
| TG 37:1 | 423.97 | 8.73 | 3.39E-10 |
| LPC 22:5 | 0.01 | -6.14 | 3.39E-10 |
| Hex2Cer 41:1;2O | 41.20 | 5.36 | 3.39E-10 |
| PI 37:4 | 38.73 | 5.28 | 3.39E-10 |
| PC 38:5 | 0.05 | -4.20 | 3.39E-10 |
| PE O-34:2 | 17.71 | 4.15 | 3.39E-10 |
| PE O-34:3 | 16.51 | 4.05 | 3.39E-10 |
| Cer 35:0;2O | 0.16 | -2.65 | 3.43E-10 |
| PC 40:6 | 0.06 | -4.02 | 3.71E-10 |
| PC 36:5 | 0.02 | -5.69 | 4.61E-10 |
| DG 36:5 | 39.39 | 5.30 | 6.40E-10 |
| TG 40:0 | 57.44 | 5.84 | 8.14E-10 |
| TG 44:1 | 39.55 | 5.31 | 8.14E-10 |
| PE O-35:2 | 25.33 | 4.66 | 8.14E-10 |
| LNAPE N-36:0 | 7.58 | 2.92 | 8.75E-10 |
| DG 24:0 | 208.53 | 7.70 | 9.98E-10 |
| TG 44:2 | 41.98 | 5.39 | 9.98E-10 |
| DG 32:1 | 20.28 | 4.34 | 1.03E-09 |
| PE 32:0 | 19.23 | 4.27 | 1.03E-09 |
| PC 38:3 | 0.07 | -3.93 | 1.12E-09 |
| PC 36:1 | 0.17 | -2.58 | 1.24E-09 |
| LPC 16:0 | 0.06 | -4.10 | 1.26E-09 |
| LPC 18:0 | 0.03 | -4.95 | 1.35E-09 |
| PC O-36:5 | 0.01 | -6.19 | 1.45E-09 |
| LPC 18:3 | 0.02 | -5.38 | 1.49E-09 |
| PE 35:1 | 41.46 | 5.37 | 1.49E-09 |
| TG 42:1 | 45.89 | 5.52 | 1.61E-09 |
| PC 40:5 | 0.05 | -4.23 | 1.61E-09 |
| TG 38:3 | 915.45 | 9.84 | 1.70E-09 |
| TG 38:0 | 62.00 | 5.95 | 1.70E-09 |
| TG 33:0 | 1284.10 | 10.33 | 1.75E-09 |
| TG 40:1 | 101.46 | 6.66 | 1.75E-09 |
| SM 34:2;2O | 0.06 | -4.05 | 1.75E-09 |
| PC 33:0 | 7.52 | 2.91 | 1.76E-09 |
| PE O-40:6 | 7.37 | 2.88 | 1.76E-09 |
| TG 36:2 | 1934.80 | 10.92 | 1.78E-09 |
| LPC 19:0 | 0.13 | -2.90 | 1.78E-09 |
| TG 35:1 | 790.42 | 9.63 | 2.07E-09 |
| PE 36:4 | 29.24 | 4.87 | 2.07E-09 |
| PC 34:3 | 0.07 | -3.94 | 2.26E-09 |
| TG 38:1 | 139.98 | 7.13 | 3.04E-09 |
| Cer 34:1;2O | 152.53 | 7.25 | 3.23E-09 |
| DG 28:1 | 194.29 | 7.60 | 3.34E-09 |
| TG 44:0 | 0.06 | -4.05 | 3.47E-09 |
| LNAPE N-34:0 | 6.82 | 2.77 | 3.48E-09 |
| PC 37:2 | 0.13 | -2.91 | 3.65E-09 |
| PE O-36:3 | 19.46 | 4.28 | 3.70E-09 |
| TG 51:3 | 89.23 | 6.48 | 3.86E-09 |
| TG 46:5 | 12.07 | 3.59 | 3.86E-09 |
| PE O-35:3 | 6.44 | 2.69 | 4.03E-09 |
| PE 34:1 | 20.54 | 4.36 | 5.15E-09 |
| LPC O-16:0 | 0.03 | -5.02 | 5.24E-09 |
| PC 38:2 | 0.17 | -2.54 | 7.13E-09 |
| TG 39:1 | 108.36 | 6.76 | 8.18E-09 |
| DG 26:0 | 69.33 | 6.12 | 8.69E-09 |
| DG 30:1 | 64.86 | 6.02 | 8.69E-09 |
| TG 48:2 | 21.83 | 4.45 | 8.69E-09 |
| TG 43:1 | 52.39 | 5.71 | 9.64E-09 |
| TG 41:2 | 49.67 | 5.63 | 9.76E-09 |
| DG 26:1 | 98.65 | 6.62 | 1.01E-08 |
| LPC O-16:1 | 0.06 | -3.95 | 1.18E-08 |
| PE O-33:2 | 11.98 | 3.58 | 1.21E-08 |
| TG 35:0 | 224.25 | 7.81 | 1.22E-08 |
| TG 44:4 | 29.12 | 4.86 | 1.36E-08 |
| PE 36:1 | 15.97 | 4.00 | 1.43E-08 |
| PC 35:0 | 4.34 | 2.12 | 1.49E-08 |
| PE 40:8 | 7.33 | 2.87 | 1.82E-08 |
| DG 36:3 | 8.15 | 3.03 | 1.86E-08 |
| TG 48:4 | 5.89 | 2.56 | 2.12E-08 |
| DG 38:0 | 0.05 | -4.28 | 2.31E-08 |
| LNAPE N-36:1 | 0.03 | -4.92 | 2.41E-08 |
| PC 36:4 | 0.04 | -4.57 | 2.55E-08 |
| PE 40:6 | 27.62 | 4.79 | 2.77E-08 |
| LPC 22:4 | 0.00 | -8.30 | 2.77E-08 |
| LPC 18:2 | 0.02 | -5.90 | 2.80E-08 |
| PE 35:2 | 7.65 | 2.94 | 3.00E-08 |
| DG 28:0 | 56.47 | 5.82 | 3.01E-08 |
| TG 48:5 | 7.99 | 3.00 | 3.01E-08 |
| DG 36:4 | 14.60 | 3.87 | 3.24E-08 |
| TG 41:1 | 42.48 | 5.41 | 3.26E-08 |
| TG 42:3 | 67.54 | 6.08 | 3.31E-08 |
| TG 45:2 | 10.55 | 3.40 | 3.33E-08 |
| TG 44:3 | 26.99 | 4.75 | 3.87E-08 |
| PE O-36:4 | 4.69 | 2.23 | 4.12E-08 |
| LPC 22:6 | 0.03 | -5.07 | 4.66E-08 |
| DG 38:5 | 13.09 | 3.71 | 4.69E-08 |
| PC O-34:1 | 0.11 | -3.17 | 4.72E-08 |
| TG 37:0 | 86.89 | 6.44 | 4.78E-08 |
| PC 37:4 | 0.06 | -4.16 | 4.87E-08 |
| TG 42:2 | 30.21 | 4.92 | 4.96E-08 |
| PE O-32:2 | 8.41 | 3.07 | 5.14E-08 |
| Cer 41:2;2O | 15.04 | 3.91 | 5.44E-08 |
| PC 32:2 | 0.04 | -4.80 | 6.09E-08 |
| PC O-36:4 | 0.06 | -4.03 | 6.18E-08 |
| TG 54:4 | 4.37 | 2.13 | 7.10E-08 |
| PE 36:3 | 5.54 | 2.47 | 7.55E-08 |
| TG 53:2 | 7.95 | 2.99 | 8.57E-08 |
| Cer 41:1;2O | 0.09 | -3.50 | 8.66E-08 |
| LNAPE N-36:2 | 0.01 | -6.72 | 8.70E-08 |
| PE 30:0 | 26.42 | 4.72 | 8.91E-08 |
| PE 33:1 | 3.98 | 1.99 | 8.91E-08 |
| LPC 15:0 | 0.03 | -5.23 | 9.24E-08 |
| TG 40:2 | 657.00 | 9.36 | 1.01E-07 |
| LPC 24:0 | 0.02 | -5.78 | 1.10E-07 |
| Cer 41:0;3O | 0.07 | -3.85 | 1.10E-07 |
| TG 43:3 | 8.47 | 3.08 | 1.10E-07 |
| PC 39:4 | 0.19 | -2.40 | 1.10E-07 |
| PE 32:1 | 42.44 | 5.41 | 1.13E-07 |
| PC 34:5 | 0.06 | -4.02 | 1.13E-07 |
| TG 62:3 | 10.54 | 3.40 | 1.31E-07 |
| LPC 18:1 | 0.06 | -4.08 | 1.32E-07 |
| TG 56:2 | 5.19 | 2.38 | 1.37E-07 |
| PC O-40:8 | 0.05 | -4.22 | 1.40E-07 |
| CAR 18:1 | 0.06 | -4.00 | 1.40E-07 |
| TG 45:3 | 4.89 | 2.29 | 1.60E-07 |
| PC 34:2 | 86.27 | 6.43 | 1.67E-07 |
| ST 27:1;O;S | 0.15 | -2.70 | 1.69E-07 |
| PC 35:5 | 0.05 | -4.44 | 2.28E-07 |
| DG 36:2 | 5.43 | 2.44 | 2.30E-07 |
| TG 54:2 | 6.87 | 2.78 | 2.60E-07 |
| PE O-38:2 | 9.68 | 3.27 | 2.66E-07 |
| TG 50:3 | 0.22 | -2.17 | 3.05E-07 |
| TG 36:1 | 1079.40 | 10.08 | 3.25E-07 |
| LPC 16:1 | 0.10 | -3.38 | 3.36E-07 |
| PI 38:4 | 0.07 | -3.79 | 4.16E-07 |
| CAR 16:0 | 0.09 | -3.48 | 4.21E-07 |
| TG 38:2 | 802.22 | 9.65 | 4.26E-07 |
| TG 36:0 | 44.59 | 5.48 | 4.34E-07 |
| TG 58:2 | 7.94 | 2.99 | 4.41E-07 |
| PE O-36:1 | 7.80 | 2.96 | 4.49E-07 |
| PS 38:4 | 8.70 | 3.12 | 4.59E-07 |
| TG 48:3 | 5.88 | 2.56 | 4.66E-07 |
| PI 40:5 | 0.20 | -2.31 | 4.73E-07 |
| LPC 20:5 | 0.01 | -6.82 | 4.97E-07 |
| PE O-40:2 | 24.26 | 4.60 | 5.02E-07 |
| SM 42:4;2O | 0.09 | -3.54 | 5.02E-07 |
| TG 53:1 | 5.45 | 2.45 | 5.02E-07 |
| PC 39:6 | 0.19 | -2.37 | 5.66E-07 |
| LPC 19:1 | 0.08 | -3.71 | 5.96E-07 |
| DG 34:4 | 17.80 | 4.15 | 6.58E-07 |
| TG 41:0 | 8.91 | 3.16 | 7.12E-07 |
| Hex2Cer 34:1;2O | 5.88 | 2.56 | 7.69E-07 |
| TG 43:0 | 5.73 | 2.52 | 7.69E-07 |
| TG 46:0 | 22.84 | 4.51 | 8.17E-07 |
| TG 60:1 | 157.82 | 7.30 | 9.26E-07 |
| LPC 20:3 | 0.01 | -6.94 | 9.29E-07 |
| PE O-38:5 | 2.56 | 1.35 | 9.41E-07 |
| LPC 20:4 | 0.01 | -6.65 | 9.92E-07 |
| Cer 38:1;2O | 0.18 | -2.49 | 1.29E-06 |
| CAR 18:0 | 0.11 | -3.15 | 1.37E-06 |
| PI 36:1 | 5.11 | 2.35 | 1.37E-06 |
| LPC O-18:1 | 0.04 | -4.69 | 1.40E-06 |
| LNAPE N-37:2 | 0.02 | -5.63 | 1.40E-06 |
| DG 32:0 | 5.05 | 2.34 | 1.40E-06 |
| LPE 20:4 | 0.02 | -5.84 | 1.43E-06 |
| PE O-32:1 | 4.91 | 2.30 | 1.60E-06 |
| TG 55:3 | 4.83 | 2.27 | 1.63E-06 |
| TG 59:3 | 6.57 | 2.72 | 1.71E-06 |
| PC O-38:5 | 0.03 | -4.89 | 1.79E-06 |
| PE O-35:4 | 4.54 | 2.18 | 1.84E-06 |
| LPC O-18:0 | 0.01 | -6.61 | 1.90E-06 |
| TG 58:3 | 4.89 | 2.29 | 1.90E-06 |
| PE 34:3 | 4.45 | 2.15 | 2.60E-06 |
| PC O-39:7 | 0.08 | -3.63 | 2.80E-06 |
| TG 52:2 | 3.79 | 1.92 | 2.86E-06 |
| PC O-37:5 | 0.05 | -4.21 | 3.00E-06 |
| TG 45:1 | 8.23 | 3.04 | 3.08E-06 |
| TG 49:2 | 3.59 | 1.84 | 3.09E-06 |
| SM 40:3;2O | 0.11 | -3.23 | 3.12E-06 |
| PE O-37:2 | 9.03 | 3.18 | 3.29E-06 |
| DG 30:0 | 14.05 | 3.81 | 3.29E-06 |
| PE O-38:4 | 3.66 | 1.87 | 3.75E-06 |
| PE-Cer 34:0;2O | 0.13 | -2.98 | 3.93E-06 |
| PE 33:2 | 4.22 | 2.08 | 3.98E-06 |
| Hex2Cer 42:1;2O | 0.10 | -3.39 | 4.01E-06 |
| PC O-40:7 | 0.09 | -3.53 | 4.07E-06 |
| PC O-34:2 | 0.19 | -2.42 | 4.11E-06 |
| PE 36:2 | 4.49 | 2.17 | 4.25E-06 |
| PC O-38:4 | 0.08 | -3.69 | 4.41E-06 |
| CAR 15:0 | 0.19 | -2.36 | 4.88E-06 |
| TG 39:0 | 27.02 | 4.76 | 4.94E-06 |
| TG 47:2 | 4.85 | 2.28 | 4.99E-06 |
| PI 36:4 | 0.18 | -2.44 | 5.21E-06 |
| DG 34:3 | 11.74 | 3.55 | 5.28E-06 |
| PE O-33:1 | 3.53 | 1.82 | 5.45E-06 |
| LNAPE N-35:2 | 0.16 | -2.68 | 6.47E-06 |
| PE 38:3 | 7.92 | 2.99 | 6.65E-06 |
| PE-Cer 42:3;2O | 3.29 | 1.72 | 6.83E-06 |
| TG 50:4 | 3.99 | 2.00 | 7.01E-06 |
| TG 60:2 | 11.77 | 3.56 | 7.10E-06 |
| TG 62:2 | 18.27 | 4.19 | 7.29E-06 |
| PC 40:7 | 0.26 | -1.92 | 8.08E-06 |
| TG 43:2 | 13.21 | 3.72 | 8.30E-06 |
| LPC O-17:1 | 0.19 | -2.42 | 8.30E-06 |
| Cer 42:2;2O | 0.16 | -2.62 | 8.65E-06 |
| TG 57:1 | 6.80 | 2.77 | 9.32E-06 |
| LNAPE N-38:3 | 0.03 | -4.94 | 9.58E-06 |
| PC 35:4 | 0.05 | -4.27 | 9.58E-06 |
| LNAPE N-38:2 | 0.06 | -4.11 | 9.58E-06 |
| PI 38:3 | 0.23 | -2.10 | 9.58E-06 |
| PI 38:5 | 0.22 | -2.18 | 1.02E-05 |
| LNAPE N-34:2 | 0.08 | -3.63 | 1.03E-05 |
| TG 34:0 | 0.02 | -5.74 | 1.07E-05 |
| CAR 14:1 | 0.18 | -2.47 | 1.19E-05 |
| TG 46:3 | 10.32 | 3.37 | 1.21E-05 |
| TG 61:2 | 29.20 | 4.87 | 1.35E-05 |
| TG 60:3 | 10.43 | 3.38 | 1.51E-05 |
| TG 42:0 | 5.15 | 2.37 | 1.51E-05 |
| PE O-40:3 | 8.10 | 3.02 | 1.56E-05 |
| PE O-33:3 | 2.89 | 1.53 | 1.64E-05 |
| DG 34:2 | 53.57 | 5.74 | 1.75E-05 |
| PC O-40:6 | 0.14 | -2.83 | 1.75E-05 |
| PE O-37:3 | 5.36 | 2.42 | 1.81E-05 |
| PC 29:0 | 4.70 | 2.23 | 2.15E-05 |
| LNAPE N-37:3 | 0.11 | -3.20 | 2.51E-05 |
| CAR 14:0 | 0.16 | -2.67 | 2.62E-05 |
| TG 55:2 | 7.05 | 2.82 | 2.84E-05 |
| SM 34:0;2O | 8.46 | 3.08 | 3.00E-05 |
| PE-NMe 35:4 | 57.91 | 5.86 | 3.01E-05 |
| PC O-36:3 | 0.26 | -1.96 | 3.13E-05 |
| PE 38:4 | 5.64 | 2.50 | 3.31E-05 |
| TG 58:1 | 21.06 | 4.40 | 3.32E-05 |
| Cer 34:0;3O | 0.26 | -1.94 | 3.96E-05 |
| LPC 14:0 | 0.15 | -2.71 | 4.49E-05 |
| LPC 22:0 | 0.19 | -2.41 | 4.76E-05 |
| PG 36:2 | 4.30 | 2.10 | 4.99E-05 |
| CAR 16:1 | 0.24 | -2.07 | 5.12E-05 |
| PE-Cer 40:2;2O | 4.52 | 2.18 | 5.29E-05 |
| TG 46:1 | 4.69 | 2.23 | 5.30E-05 |
| PC 34:0 | 19.43 | 4.28 | 5.49E-05 |
| PE 32:2 | 7.72 | 2.95 | 5.75E-05 |
| PE O-40:8 | 2.43 | 1.28 | 5.79E-05 |
| TG 54:5 | 3.45 | 1.79 | 5.87E-05 |
| TG 56:1 | 5.75 | 2.52 | 6.13E-05 |
| PC O-40:5 | 0.16 | -2.64 | 6.49E-05 |
| PC 37:5 | 0.26 | -1.94 | 6.64E-05 |
| LPE 22:6 | 0.05 | -4.32 | 7.65E-05 |
| PE O-40:4 | 4.52 | 2.18 | 7.92E-05 |
| PC 40:8 | 0.23 | -2.11 | 8.19E-05 |
| TG 50:0 | 0.08 | -3.61 | 9.04E-05 |
| PE O-37:7 | 0.28 | -1.84 | 9.07E-05 |
| TG 53:3 | 3.98 | 1.99 | 9.08E-05 |
| TG 48:6 | 7.65 | 2.93 | 9.39E-05 |
| TG 61:1 | 8.51 | 3.09 | 9.62E-05 |
| PA 34:2 | 0.21 | -2.27 | 9.70E-05 |
| SM 38:2;2O | 0.26 | -1.97 | 1.06E-04 |
| PE 31:1 | 4.23 | 2.08 | 1.45E-04 |
| DG 38:6 | 12.35 | 3.63 | 1.48E-04 |
| PE 39:5 | 6.03 | 2.59 | 1.55E-04 |
| TG 60:0 | 50.13 | 5.65 | 1.95E-04 |
| PC O-42:4 | 0.22 | -2.17 | 2.03E-04 |
| FFA(24:2) | 0.15 | -2.73 | 2.05E-04 |
| Cer 34:0;2O | 2.55 | 1.35 | 2.66E-04 |
| SM 34:3;2O | 0.04 | -4.49 | 2.71E-04 |
| PE O-38:3 | 16.88 | 4.08 | 2.72E-04 |
| PC 24:0 | 10.01 | 3.32 | 2.72E-04 |
| PC O-33:6 | 3.12 | 1.64 | 2.74E-04 |
| PC O-42:6 | 0.15 | -2.71 | 2.77E-04 |
| TG 52:5 | 3.17 | 1.66 | 2.80E-04 |
| PC 39:5 | 0.17 | -2.60 | 2.87E-04 |
| PC 37:6 | 0.15 | -2.77 | 2.94E-04 |
| TG 49:3 | 2.74 | 1.45 | 3.02E-04 |
| PC 20:0 | 4.40 | 2.14 | 3.15E-04 |
| PI 36:2 | 2.61 | 1.38 | 3.15E-04 |
| PE O-32:3 | 3.98 | 1.99 | 3.16E-04 |
| PE 31:0 | 5.92 | 2.56 | 3.38E-04 |
| PC 33:2 | 0.18 | -2.48 | 3.90E-04 |
| CAR 12:0 | 0.25 | -2.00 | 4.68E-04 |
| PC 36:6 | 0.23 | -2.13 | 4.71E-04 |
| LPE 18:3 | 0.16 | -2.64 | 4.73E-04 |
| ST 24:1;O4 | 5.85 | 2.55 | 4.92E-04 |
| PG 36:1 | 3.10 | 1.63 | 5.00E-04 |
| TG 52:0 | 7.30 | 2.87 | 6.70E-04 |
| LNAPE N-36:3 | 0.05 | -4.44 | 6.76E-04 |
| Cer 42:1;3O | 0.21 | -2.28 | 6.86E-04 |
| LPE 22:5 | 0.18 | -2.48 | 6.92E-04 |
| Cer 36:0;2O | 4.18 | 2.06 | 6.97E-04 |
| PC 31:1 | 2.48 | 1.31 | 8.12E-04 |
| LPE 20:3 | 0.15 | -2.74 | 9.19E-04 |
| LPE 20:5 | 0.16 | -2.64 | 9.19E-04 |
| PC 30:2 | 0.13 | -2.92 | 9.59E-04 |
| PE O-33:4 | 3.69 | 1.88 | 9.85E-04 |
| LNAPE N-38:4 | 0.09 | -3.50 | 1.03E-03 |
| TG 56:0 | 12.62 | 3.66 | 1.16E-03 |
| TG 32:0 | 0.06 | -3.98 | 1.17E-03 |
| SM 32:2;2O | 0.18 | -2.49 | 1.19E-03 |
| PE O-34:1 | 2.64 | 1.40 | 1.21E-03 |
| LPC O-24:1 | 0.26 | -1.97 | 1.29E-03 |
| PE O-42:9 | 2.89 | 1.53 | 1.38E-03 |
| PE O-38:8 | 0.38 | -1.38 | 1.48E-03 |
| PE-Cer 36:1;2O | 0.37 | -1.45 | 1.51E-03 |
| PC 26:0 | 4.32 | 2.11 | 1.53E-03 |
| PC O-34:0 | 0.44 | -1.19 | 1.57E-03 |
| PC 40:9 | 0.31 | -1.69 | 1.63E-03 |
| FFA(24:3) | 0.28 | -1.82 | 1.77E-03 |
| PE 34:4 | 3.39 | 1.76 | 1.85E-03 |
| FFA(24:1) | 0.34 | -1.57 | 1.85E-03 |
| SM 36:3;2O | 0.18 | -2.47 | 2.06E-03 |
| TG 26:0 | 7.60 | 2.93 | 2.22E-03 |
| PE 40:7 | 2.46 | 1.30 | 2.27E-03 |
| SM 42:1;3O | 0.34 | -1.56 | 2.45E-03 |
| TG 57:3 | 4.49 | 2.17 | 3.21E-03 |
| TG 46:2 | 0.26 | -1.95 | 3.35E-03 |
| LPE 18:2 | 0.24 | -2.05 | 4.40E-03 |
| Hex2Cer 42:2;2O | 0.43 | -1.21 | 4.42E-03 |
| TG 49:0 | 7.90 | 2.98 | 4.77E-03 |
| LPC 20:2 | 0.17 | -2.57 | 5.64E-03 |
| TG 56:4 | 2.35 | 1.23 | 6.21E-03 |
| TG 52:1 | 5.13 | 2.36 | 6.67E-03 |
| PC O-32:0 | 0.19 | -2.36 | 7.44E-03 |
| TG 60:4 | 3.08 | 1.62 | 7.54E-03 |
| DG 36:1 | 7.08 | 2.82 | 7.79E-03 |
| PC O-44:6 | 0.33 | -1.59 | 8.35E-03 |
| LNAPE N-40:3 | 0.26 | -1.93 | 9.85E-03 |
| PC O-35:1 | 0.12 | -3.01 | 1.18E-02 |
| TG 53:0 | 5.42 | 2.44 | 1.45E-02 |
| TG 48:0 | 3.70 | 1.89 | 1.48E-02 |
| LPC 20:0 | 0.15 | -2.78 | 1.50E-02 |
| TG 51:5 | 2.07 | 1.05 | 1.76E-02 |
| FFA(18:2) | 0.33 | -1.62 | 1.78E-02 |
| PA 38:6 | 0.33 | -1.60 | 1.82E-02 |
| Hex2Cer 32:1;2O | 0.41 | -1.28 | 1.87E-02 |
| PC O-44:5 | 0.26 | -1.96 | 1.97E-02 |
| SM 42:5;2O | 0.28 | -1.82 | 2.03E-02 |
| PC O-35:2 | 2.21 | 1.15 | 2.28E-02 |
| TG 58:11 | 0.50 | -1.01 | 2.35E-02 |
| PC O-37:7 | 2.99 | 1.58 | 2.42E-02 |
| Cer 40:2;2O | 0.48 | -1.06 | 2.91E-02 |
| PE O-42:4 | 2.13 | 1.09 | 3.06E-02 |
| LPC O-24:0 | 0.30 | -1.74 | 3.23E-02 |
| PE O-42:5 | 0.27 | -1.91 | 3.36E-02 |
| FFA(20:2) | 0.45 | -1.17 | 3.59E-02 |
| LNAPE N-40:6 | 0.29 | -1.77 | 4.26E-02 |
| SM 36:4;2O | 0.18 | -2.47 | 4.28E-02 |

S6. Differential lipids in maternal plasma compared to lamb plasma

| **Lipid Name** | **FC** | **log2(FC)** | **Adjusted P** |
| --- | --- | --- | --- |
| TG 40:1 | 0.01 | -6.31 | 5.24E-08 |
| TG 40:0 | 0.02 | -5.51 | 5.24E-08 |
| TG 44:2 | 0.02 | -5.36 | 5.24E-08 |
| TG 44:1 | 0.03 | -5.09 | 5.24E-08 |
| TG 42:1 | 0.02 | -5.91 | 8.36E-08 |
| TG 42:0 | 0.01 | -6.78 | 1.43E-07 |
| TG 42:2 | 0.01 | -6.35 | 1.43E-07 |
| TG 41:1 | 0.03 | -4.87 | 1.56E-07 |
| LPC 22:6 | 0.35 | -1.50 | 3.56E-07 |
| TG 40:2 | 0.02 | -5.46 | 4.20E-07 |
| SM 29:1;2O | 0.19 | -2.36 | 4.20E-07 |
| TG 41:0 | 0.02 | -5.35 | 5.81E-07 |
| LPA 16:0 | 0.41 | -1.30 | 6.51E-07 |
| TG 44:0 | 0.06 | -4.03 | 7.14E-07 |
| TG 50:6 | 0.17 | -2.59 | 7.50E-07 |
| PC 30:0 | 0.22 | -2.20 | 8.76E-07 |
| TG 38:0 | 0.03 | -4.85 | 9.15E-07 |
| TG 43:0 | 0.05 | -4.20 | 1.22E-06 |
| TG 44:3 | 0.03 | -5.29 | 1.23E-06 |
| TG 46:1 | 0.05 | -4.34 | 1.49E-06 |
| CAR 14:0 | 0.23 | -2.11 | 1.57E-06 |
| LPE 22:6 | 0.21 | -2.23 | 1.62E-06 |
| TG 35:0 | 0.04 | -4.54 | 2.35E-06 |
| TG 36:0 | 0.01 | -6.24 | 3.09E-06 |
| SM 42:3;2O | 0.46 | -1.12 | 3.41E-06 |
| PE O-33:3 | 5.21 | 2.38 | 3.57E-06 |
| TG 46:0 | 0.22 | -2.16 | 4.04E-06 |
| TG 38:1 | 0.02 | -5.71 | 4.30E-06 |
| TG 42:3 | 0.02 | -5.32 | 4.30E-06 |
| SM 38:1;2O | 0.36 | -1.46 | 4.43E-06 |
| TG 43:3 | 0.11 | -3.17 | 4.61E-06 |
| PE-Cer 34:1;2O | 0.45 | -1.16 | 4.84E-06 |
| TG 48:5 | 0.15 | -2.73 | 5.45E-06 |
| Cer 39:1;2O | 0.25 | -1.98 | 5.45E-06 |
| HexCer 34:1;2O | 0.30 | -1.72 | 5.45E-06 |
| PE-Cer 42:3;2O | 0.33 | -1.61 | 5.45E-06 |
| LPC 14:0 | 0.23 | -2.12 | 6.58E-06 |
| PI 40:6 | 0.20 | -2.35 | 6.60E-06 |
| SM 28:0;2O | 0.17 | -2.53 | 6.95E-06 |
| TG 45:1 | 0.09 | -3.47 | 7.08E-06 |
| PC 30:1 | 0.35 | -1.51 | 1.01E-05 |
| LPE O-16:1 | 0.44 | -1.17 | 1.01E-05 |
| LPC 20:0 | 0.46 | -1.11 | 1.01E-05 |
| TG 48:1 | 0.16 | -2.66 | 1.03E-05 |
| CAR 16:0 | 0.28 | -1.84 | 1.03E-05 |
| TG 52:7 | 0.29 | -1.78 | 1.03E-05 |
| Cer 41:1;2O | 0.33 | -1.62 | 1.03E-05 |
| PI 38:4 | 0.38 | -1.38 | 1.03E-05 |
| TG 45:2 | 0.13 | -3.00 | 1.05E-05 |
| TG 39:1 | 0.04 | -4.59 | 1.18E-05 |
| PC 30:2 | 0.16 | -2.64 | 1.18E-05 |
| FFA(20:4) Arachidonic acid | 0.41 | -1.28 | 1.18E-05 |
| TG 43:1 | 0.05 | -4.30 | 1.31E-05 |
| PE O-33:2 | 3.29 | 1.72 | 1.39E-05 |
| CAR 15:0 | 0.46 | -1.11 | 1.46E-05 |
| SM 32:1;2O | 0.39 | -1.35 | 1.58E-05 |
| SM 28:1;2O | 0.13 | -2.93 | 1.71E-05 |
| TG 48:3 | 0.18 | -2.45 | 1.71E-05 |
| PC O-22:0 | 0.38 | -1.40 | 1.78E-05 |
| TG 46:2 | 0.03 | -5.04 | 1.93E-05 |
| PE O-34:5 | 3.13 | 1.65 | 2.01E-05 |
| TG 34:0 | 0.03 | -5.09 | 2.15E-05 |
| PI 36:4 | 0.14 | -2.82 | 2.22E-05 |
| SM 27:1;2O | 0.26 | -1.93 | 2.59E-05 |
| PE O-40:8 | 0.40 | -1.31 | 2.59E-05 |
| LPC 22:0 | 0.32 | -1.66 | 2.62E-05 |
| TG 39:0 | 0.01 | -6.20 | 2.66E-05 |
| LPE 20:4 | 0.34 | -1.55 | 2.78E-05 |
| LPC 21:0 | 0.37 | -1.43 | 2.87E-05 |
| TG 37:0 | 0.03 | -4.86 | 3.18E-05 |
| TG 53:4 | 3.04 | 1.61 | 3.37E-05 |
| TG 48:2 | 0.26 | -1.93 | 3.60E-05 |
| TG 48:0 | 0.27 | -1.90 | 3.69E-05 |
| PE-Cer 42:2;2O | 0.44 | -1.18 | 3.72E-05 |
| PE 33:0 | 2.81 | 1.49 | 4.76E-05 |
| SM 40:2;2O | 0.45 | -1.16 | 5.02E-05 |
| SM 42:4;2O | 0.43 | -1.22 | 5.11E-05 |
| TG 46:3 | 0.04 | -4.48 | 5.46E-05 |
| HexCer 42:2;2O | 0.38 | -1.41 | 5.46E-05 |
| TG 56:7 | 0.38 | -1.39 | 5.46E-05 |
| LPC 15:1 | 0.43 | -1.21 | 5.62E-05 |
| PE O-18:1 | 0.43 | -1.21 | 5.62E-05 |
| PC 42:10 | 0.45 | -1.15 | 5.65E-05 |
| TG 46:4 | 0.05 | -4.37 | 6.65E-05 |
| TG 34:1 | 0.10 | -3.37 | 7.10E-05 |
| TG 48:6 | 0.10 | -3.31 | 7.10E-05 |
| TG 58:8 | 0.34 | -1.55 | 7.27E-05 |
| PE O-33:4 | 14.26 | 3.83 | 7.27E-05 |
| PI 34:2 | 0.25 | -1.99 | 8.33E-05 |
| Cer 43:1;2O | 0.40 | -1.31 | 8.93E-05 |
| SM 36:3;2O | 0.48 | -1.07 | 8.95E-05 |
| TG 52:6 | 0.33 | -1.61 | 1.01E-04 |
| TG 44:4 | 0.08 | -3.65 | 1.06E-04 |
| PE O-31:1 | 3.71 | 1.89 | 1.06E-04 |
| TG 50:5 | 0.31 | -1.71 | 1.07E-04 |
| PC 28:0 | 0.25 | -1.98 | 1.16E-04 |
| TG 43:2 | 0.08 | -3.56 | 1.18E-04 |
| PI 38:3 | 0.48 | -1.07 | 1.25E-04 |
| TG 50:4 | 0.34 | -1.56 | 1.39E-04 |
| PG 36:2 | 4.32 | 2.11 | 1.40E-04 |
| LPC 24:5 | 0.40 | -1.31 | 1.44E-04 |
| TG 54:6 | 0.47 | -1.09 | 1.44E-04 |
| LPC 36:2 | 3.87 | 1.95 | 1.44E-04 |
| SM 39:1;2O | 0.33 | -1.60 | 1.57E-04 |
| PC O-36:2 | 3.36 | 1.75 | 1.57E-04 |
| LPE 18:1 | 0.39 | -1.34 | 1.58E-04 |
| TG 38:2 | 0.04 | -4.65 | 1.60E-04 |
| PC 39:1 | 0.49 | -1.04 | 1.64E-04 |
| PC O-23:1 | 0.36 | -1.47 | 1.67E-04 |
| TG 50:2 | 0.30 | -1.71 | 1.72E-04 |
| TG 48:4 | 0.23 | -2.13 | 1.84E-04 |
| TG 41:2 | 0.13 | -2.89 | 1.89E-04 |
| Cer 40:1;2O | 0.29 | -1.79 | 1.95E-04 |
| PC 32:0 | 0.45 | -1.16 | 1.95E-04 |
| PE 40:7 | 0.34 | -1.57 | 2.13E-04 |
| SM 30:1;2O | 0.29 | -1.77 | 2.37E-04 |
| SM 32:0;2O | 0.30 | -1.75 | 2.37E-04 |
| TG 47:2 | 0.29 | -1.81 | 2.69E-04 |
| PC 29:0 | 0.31 | -1.70 | 2.69E-04 |
| Cer 38:0;3O | 0.23 | -2.11 | 2.75E-04 |
| TG 36:1 | 0.05 | -4.20 | 2.82E-04 |
| PI 38:5 | 0.35 | -1.52 | 2.88E-04 |
| LPE 22:5 | 0.42 | -1.24 | 2.88E-04 |
| CAR 18:1 | 0.39 | -1.37 | 2.88E-04 |
| Cer 32:1;2O | 0.30 | -1.73 | 2.90E-04 |
| PE O-35:6 | 2.74 | 1.45 | 3.06E-04 |
| TG 40:3 | 0.11 | -3.21 | 3.09E-04 |
| Cer 38:1;2O | 0.29 | -1.81 | 3.46E-04 |
| TG 42:4 | 0.18 | -2.48 | 3.52E-04 |
| TG 46:5 | 0.18 | -2.48 | 3.52E-04 |
| PC 27:0 | 0.33 | -1.62 | 3.52E-04 |
| PC 40:3 | 2.53 | 1.34 | 3.52E-04 |
| LNAPE N-40:3 | 4.63 | 2.21 | 3.52E-04 |
| PE O-38:2 | 2.99 | 1.58 | 3.58E-04 |
| PE O-32:1 | 2.63 | 1.40 | 3.65E-04 |
| TG 60:10 | 0.20 | -2.35 | 3.97E-04 |
| TG 45:0 | 0.14 | -2.84 | 4.16E-04 |
| TG 54:7 | 0.38 | -1.41 | 4.20E-04 |
| TG 50:3 | 0.34 | -1.55 | 4.27E-04 |
| DG 30:1 | 0.24 | -2.05 | 4.68E-04 |
| PC 30:3 | 0.23 | -2.11 | 4.72E-04 |
| Cer 36:2;2O | 0.40 | -1.31 | 4.87E-04 |
| CAR 16:1 | 0.42 | -1.27 | 5.01E-04 |
| PC 42:4 | 0.45 | -1.15 | 5.26E-04 |
| SM 40:4;2O | 0.47 | -1.08 | 5.26E-04 |
| TG 60:11 | 0.23 | -2.15 | 5.26E-04 |
| PC 28:1 | 0.27 | -1.90 | 5.32E-04 |
| SM 36:1;3O | 0.35 | -1.53 | 5.32E-04 |
| PC O-35:2 | 3.26 | 1.71 | 5.39E-04 |
| LNAPE N-40:0 | 4.03 | 2.01 | 5.54E-04 |
| Cer 42:1;2O | 0.41 | -1.29 | 5.98E-04 |
| LPC O-24:1 | 0.32 | -1.65 | 6.35E-04 |
| LPC 24:1 | 0.48 | -1.05 | 6.35E-04 |
| PE O-34:4 | 5.73 | 2.52 | 6.35E-04 |
| TG 46:6 | 0.09 | -3.51 | 6.56E-04 |
| TG 42:5 | 0.24 | -2.07 | 7.21E-04 |
| PE O-32:3 | 3.11 | 1.64 | 7.66E-04 |
| PC 31:0 | 3.07 | 1.62 | 7.76E-04 |
| SM 36:2;2O | 0.48 | -1.06 | 8.08E-04 |
| LNAPE N-32:0 | 0.31 | -1.70 | 8.19E-04 |
| PC 40:1 | 0.44 | -1.19 | 8.19E-04 |
| TG 45:3 | 0.25 | -2.03 | 8.35E-04 |
| CE 22:6 | 0.40 | -1.31 | 8.45E-04 |
| PE O-29:1 | 15.70 | 3.97 | 8.46E-04 |
| TG 58:11 | 0.34 | -1.55 | 8.59E-04 |
| ST 27:1;O;Hex | 0.39 | -1.36 | 8.59E-04 |
| TG 36:2 | 0.21 | -2.25 | 8.65E-04 |
| CerP 32:1;2O | 0.32 | -1.62 | 8.85E-04 |
| PC O-42:2 | 0.48 | -1.06 | 9.02E-04 |
| TG 30:0 | 0.22 | -2.22 | 9.14E-04 |
| TG 51:4 | 2.52 | 1.33 | 9.33E-04 |
| LPC O-14:1 | 4.06 | 2.02 | 9.52E-04 |
| PI 34:1 | 0.49 | -1.04 | 1.03E-03 |
| TG 37:1 | 0.14 | -2.79 | 1.04E-03 |
| PE O-36:2 | 2.48 | 1.31 | 1.09E-03 |
| PC 42:5 | 4.24 | 2.09 | 1.14E-03 |
| PG O-34:2 | 0.40 | -1.31 | 1.14E-03 |
| Cer 41:2;2O | 0.33 | -1.61 | 1.16E-03 |
| LPC 24:0 | 0.41 | -1.28 | 1.23E-03 |
| CAR 18:0 | 0.47 | -1.08 | 1.32E-03 |
| ST 27:1;O;S | 0.43 | -1.20 | 1.39E-03 |
| PE O-32:2 | 2.35 | 1.23 | 1.42E-03 |
| PC O-43:4 | 0.50 | -1.01 | 1.43E-03 |
| LNAPE N-34:2 | 0.24 | -2.06 | 1.46E-03 |
| SM 33:4;2O | 0.33 | -1.61 | 1.52E-03 |
| DG 32:0 | 0.32 | -1.66 | 1.54E-03 |
| PC 44:4 | 0.38 | -1.41 | 1.54E-03 |
| LNAPE N-40:1 | 7.48 | 2.90 | 1.61E-03 |
| PC 38:0 | 2.68 | 1.42 | 1.64E-03 |
| HexCer 34:2;2O | 0.29 | -1.77 | 1.69E-03 |
| PE O-36:3 | 2.87 | 1.52 | 1.72E-03 |
| PC 32:3 | 0.37 | -1.44 | 1.76E-03 |
| PE 40:3 | 4.11 | 2.04 | 1.78E-03 |
| PC 44:5 | 0.41 | -1.29 | 1.78E-03 |
| CAR 24:0 | 0.45 | -1.16 | 1.78E-03 |
| PG 33:0 | 3.99 | 2.00 | 1.91E-03 |
| TG 47:3 | 0.48 | -1.06 | 1.97E-03 |
| TG 32:0 | 0.09 | -3.52 | 2.04E-03 |
| Cer 42:3;2O | 0.38 | -1.40 | 2.06E-03 |
| TG 58:7 | 0.48 | -1.07 | 2.06E-03 |
| SM 26:1;2O | 0.28 | -1.85 | 2.20E-03 |
| DG 28:1 | 0.31 | -1.70 | 2.23E-03 |
| DG 26:0 | 0.19 | -2.39 | 2.26E-03 |
| PE-Cer 33:1;2O | 0.43 | -1.21 | 2.26E-03 |
| PC 34:5 | 0.46 | -1.13 | 2.26E-03 |
| PE O-37:7 | 4.50 | 2.17 | 2.36E-03 |
| PE O-38:8 | 3.22 | 1.69 | 2.50E-03 |
| PE O-30:1 | 3.09 | 1.63 | 2.53E-03 |
| TG 58:9 | 0.32 | -1.65 | 2.80E-03 |
| ST 24:1;O5 | 4.41 | 2.14 | 2.81E-03 |
| PE O-35:3 | 2.09 | 1.06 | 2.82E-03 |
| PE O-34:6 | 2.55 | 1.35 | 2.85E-03 |
| CAR 14:1 | 0.47 | -1.08 | 2.90E-03 |
| PE O-35:2 | 2.24 | 1.16 | 2.90E-03 |
| PC O-44:5 | 0.49 | -1.04 | 2.94E-03 |
| PE O-33:1 | 2.75 | 1.46 | 3.00E-03 |
| DG 36:0 | 2.35 | 1.24 | 3.00E-03 |
| SM 42:5;2O | 0.47 | -1.09 | 3.05E-03 |
| PC O-32:3 | 2.47 | 1.30 | 3.38E-03 |
| PI 38:6 | 0.27 | -1.91 | 3.46E-03 |
| DG 24:0 | 0.33 | -1.58 | 3.47E-03 |
| TG 47:1 | 0.24 | -2.08 | 3.52E-03 |
| Cer 42:1;3O | 0.44 | -1.18 | 3.55E-03 |
| PE O-37:3 | 3.90 | 1.96 | 3.55E-03 |
| PC 41:5 | 3.61 | 1.85 | 3.55E-03 |
| TG 38:3 | 0.25 | -1.97 | 3.69E-03 |
| TG 58:10 | 0.28 | -1.86 | 3.80E-03 |
| CerP 34:2;2O | 0.40 | -1.31 | 3.88E-03 |
| CAR 17:0 | 0.50 | -1.00 | 4.00E-03 |
| PC 42:6 | 3.44 | 1.78 | 4.02E-03 |
| TG 56:0 | 4.04 | 2.02 | 4.05E-03 |
| PE-NMe 35:4 | 0.43 | -1.23 | 4.08E-03 |
| PC 39:3 | 2.18 | 1.12 | 4.12E-03 |
| PE O-38:5 | 2.30 | 1.20 | 4.31E-03 |
| LNAPE N-37:2 | 3.16 | 1.66 | 4.46E-03 |
| SM 34:3;2O | 0.39 | -1.36 | 4.53E-03 |
| PC O-33:2 | 3.35 | 1.74 | 4.80E-03 |
| PE 33:1 | 2.25 | 1.17 | 4.89E-03 |
| PC 29:1 | 0.41 | -1.27 | 5.03E-03 |
| DG 30:0 | 0.19 | -2.42 | 5.33E-03 |
| FFA(24:2) | 0.46 | -1.13 | 5.41E-03 |
| PE O-31:2 | 4.95 | 2.31 | 5.41E-03 |
| HexCer 42:1;2O | 0.50 | -1.01 | 5.61E-03 |
| TG 51:3 | 0.27 | -1.87 | 6.17E-03 |
| LNAPE N-39:2 | 3.30 | 1.72 | 6.60E-03 |
| HexCer 40:1;2O | 0.44 | -1.20 | 6.68E-03 |
| TG 54:8 | 0.46 | -1.12 | 6.84E-03 |
| SM 30:0;2O | 0.44 | -1.19 | 7.14E-03 |
| SPB 18:1;2O | 3.98 | 1.99 | 7.16E-03 |
| TG 33:0 | 0.18 | -2.43 | 7.64E-03 |
| TG 60:12 | 0.28 | -1.83 | 7.69E-03 |
| Hex2Cer 32:1;2O | 0.39 | -1.35 | 8.20E-03 |
| SM 39:2;2O | 0.42 | -1.27 | 8.48E-03 |
| PI 36:3 | 0.49 | -1.02 | 8.58E-03 |
| DG 28:0 | 0.28 | -1.83 | 8.62E-03 |
| LNAPE N-37:1 | 3.40 | 1.77 | 8.78E-03 |
| SM 31:0;2O | 0.43 | -1.23 | 9.30E-03 |
| CAR 12:0 | 0.34 | -1.54 | 9.65E-03 |
| TG 60:9 | 0.23 | -2.12 | 9.68E-03 |
| Cer 40:2;2O | 0.30 | -1.75 | 1.00E-02 |
| PC 32:2 | 0.44 | -1.18 | 1.01E-02 |
| Cer 39:0;2O | 0.35 | -1.51 | 1.10E-02 |
| SM 42:0;3O | 2.87 | 1.52 | 1.10E-02 |
| TG 56:8 | 0.44 | -1.20 | 1.11E-02 |
| PC O-31:1 | 2.01 | 1.01 | 1.11E-02 |
| PMeOH 34:1 | 2.87 | 1.52 | 1.14E-02 |
| PC 35:4 | 2.48 | 1.31 | 1.18E-02 |
| PC 42:3 | 0.45 | -1.16 | 1.21E-02 |
| PC 24:0 | 2.97 | 1.57 | 1.32E-02 |
| PG 40:0 | 7.19 | 2.85 | 1.40E-02 |
| LPC 26:0 | 2.51 | 1.33 | 1.51E-02 |
| PE 31:1 | 0.44 | -1.19 | 1.54E-02 |
| PE O-40:4 | 0.42 | -1.24 | 1.55E-02 |
| DG O-39:2 | 2.25 | 1.17 | 1.58E-02 |
| TG 54:9 | 0.41 | -1.29 | 1.67E-02 |
| Cer 38:2;2O | 0.35 | -1.52 | 1.68E-02 |
| TG 56:9 | 0.40 | -1.31 | 1.89E-02 |
| TG 35:1 | 0.40 | -1.34 | 1.92E-02 |
| SM 32:2;2O | 0.33 | -1.61 | 2.13E-02 |
| PE-Cer 32:1;2O | 0.41 | -1.30 | 2.13E-02 |
| TG 49:5 | 0.33 | -1.59 | 2.29E-02 |
| Cer 43:2;2O | 0.39 | -1.35 | 2.31E-02 |
| SM 40:1;2O(FA 16:0) | 0.47 | -1.08 | 2.35E-02 |
| PG 36:1 | 2.34 | 1.23 | 2.43E-02 |
| TG 47:0 | 0.43 | -1.21 | 2.74E-02 |
| LPE 17:0 | 2.38 | 1.25 | 2.80E-02 |
| PE 30:0 | 0.47 | -1.09 | 2.81E-02 |
| DG 38:6 | 0.44 | -1.19 | 3.04E-02 |
| PE-NMe2 32:1 | 0.32 | -1.62 | 3.14E-02 |
| FAHFA 26:0 | 0.38 | -1.39 | 3.19E-02 |
| FFA(24:3 | 0.42 | -1.24 | 3.23E-02 |
| PE O-42:4 | 3.04 | 1.60 | 3.53E-02 |
| CAR 14:2 | 0.46 | -1.11 | 3.65E-02 |
| PE O-38:3 | 4.83 | 2.27 | 3.71E-02 |
| CAR 18:2 | 0.49 | -1.01 | 4.91E-02 |

S7. Correlated lipids between maternal plasma, breastmilk and lamb plasma

| **Lipids** | **Correlation Coefficient** | **P Value** | **Lipid Class** |
| --- | --- | --- | --- |
| **Correlated lipids between Maternal Plasma and Breastmilk** | | | |
| PC O-44:5 | -0.76 | 2.78E-03 | Phospholipids |
| PC 34:0* | -0.61 | 0.03 | Phospholipids |
| PE 36:1* | -0.75 | 2.96E-03 | Phospholipids |
| TG 48:3* | -0.72 | 0.01 | Glycerolipids |
| TG 44:2* | -0.69 | 0.01 | Glycerolipids |
| TG 45:2* | -0.69 | 0.01 | Glycerolipids |
| DG 28:0* | -0.66 | 0.01 | Glycerolipids |
| LPC 20:3* | 0.6 | 0.03 | Phospholipids |
| PE O-18:0* | 0.62 | 0.03 | Phospholipids |
| PG 40:0* | 0.62 | 0.02 | Phospholipids |
| PE O-39:7 | 0.63 | 0.02 | Phospholipids |
| PC 28:1* | 0.67 | 0.01 | Phospholipids |
| PE O-33:2* | 0.7 | 0.01 | Phospholipids |
| PE O-39:6 | 0.64 | 0.02 | Phospholipids |
| PE O-31:1* | 0.66 | 0.01 | Phospholipids |
| PC O-33:6* | 0.75 | 2.88E-03 | Phospholipids |
| PC 40:2 | 0.82 | 6.19E-04 | Phospholipids |
| PC 26:0* | 0.92 | 6.82E-06 | Phospholipids |
| TG 60:11 | 0.76 | 2.46E-03 | Glycerolipids |
| TG 44:0 | 0.66 | 0.01 | Glycerolipids |
| TG 56:0* | 0.66 | 0.01 | Glycerolipids |
| TG 24:0* | 0.68 | 0.01 | Glycerolipids |
| TG 54:8* | 0.69 | 0.01 | Glycerolipids |
| TG 36:0* | 0.71 | 0.01 | Glycerolipids |
| DG 36:1* | 0.64 | 0.02 | Glycerolipids |
| TG 58:1* | 0.81 | 7.84E-04 | Glycerolipids |
| TG 61:2* | 0.89 | 4.18E-05 | Glycerolipids |
| Cer 32:1;2O* | 0.64 | 0.02 | Sphingolipids |
| Cer 34:1;2O* | 0.77 | 1.99E-03 | Sphingolipids |
| SM 32:2;2O | 0.75 | 3.01E-03 | Sphingolipids |
| FFA 24:3 | 0.75 | 3.21E-03 | Fatty Acyls |
| **Correlated lipids between Breastmilk and Lamb Plasma** | | | |
| LNAPE N-38:3* | 0.69 | 0.01 | Phospholipids |
| LPC 19:0* | 0.62 | 0.02 | Phospholipids |
| PC 28:0 | 0.62 | 0.02 | Phospholipids |
| PC O-34:0* | 0.83 | 4.50E-04 | Phospholipids |
| PC O-40:8* | 0.6 | 0.03 | Phospholipids |
| PE O-38:5* | 0.81 | 6.94E-04 | Phospholipids |
| PE O-39:7 | 0.68 | 0.01 | Phospholipids |
| PE O-40:2* | 0.94 | 2.35E-06 | Phospholipids |
| PE O-40:5 | 0.9 | 2.88E-05 | Phospholipids |
| PE O-40:7 | 0.73 | 4.53E-03 | Phospholipids |
| PE O-40:8* | 0.71 | 0.01 | Phospholipids |
| TG 40:3* | 0.66 | 0.01 | Glycerolipids |
| TG 45:3* | 0.81 | 7.23E-04 | Glycerolipids |
| TG 46:5* | 0.68 | 0.01 | Glycerolipids |
| TG 50:3* | 0.71 | 0.01 | Glycerolipids |
| TG 52:4 | 0.64 | 0.02 | Glycerolipids |
| TG 55:2* | 0.65 | 0.02 | Glycerolipids |
| TG 56:2* | 0.63 | 0.02 | Glycerolipids |
| TG 58:2* | 0.63 | 0.02 | Glycerolipids |
| TG 58:6 | 0.6 | 0.03 | Glycerolipids |
| TG 58:7 | 0.61 | 0.03 | Glycerolipids |
| TG 58:8 | 0.69 | 0.01 | Glycerolipids |
| TG 59:3* | 0.79 | 1.41E-03 | Glycerolipids |
| TG 60:2* | 0.71 | 0.01 | Glycerolipids |
| TG 60:3* | 0.62 | 0.02 | Glycerolipids |
| TG 62:3* | 0.7 | 0.01 | Glycerolipids |
| TG.54.5* | 0.75 | 2.86E-03 | Glycerolipids |
| DG 34:3* | 0.61 | 0.03 | Glycerolipids |
| PE-Cer 35:1;2O | 0.69 | 0.01 | Sphingolipids |
| TG 38:1* | -0.71 | 0.01 | Glycerolipids |
| TG 40:1* | -0.68 | 0.01 | Glycerolipids |
| TG 45:1* | -0.61 | 0.03 | Glycerolipids |
| **Correlated lipids between Maternal Plasma and Lamb Plasma** | | | |
| LNAPE N-34:0 | -0.75 | 3.31E-03 | Phospholipids |
| PG 36:3 | -0.62 | 0.03 | Phospholipids |
| TG 55:3 | -0.65 | 0.02 | Glycerolipids |
| SM 35:1;3O | -0.64 | 0.02 | Sphingolipids |
| PC 33:2 | 0.6 | 0.03 | Phospholipids |
| PE O-18:0 | 0.6 | 0.03 | Phospholipids |
| PC 34:1 | 0.62 | 0.02 | Phospholipids |
| PC 34:3 | 0.63 | 0.02 | Phospholipids |
| PC 36:4 | 0.63 | 0.02 | Phospholipids |
| LPC 16:1 | 0.67 | 0.01 | Phospholipids |
| PE O-36:5 | 0.7 | 0.01 | Phospholipids |
| PC 31:1 | 0.79 | 1.28E-03 | Phospholipids |
| LPC 22:6 | 0.86 | 1.43E-04 | Phospholipids |
| LNAPE N-38:5 | 0.88 | 6.90E-05 | Phospholipids |
| TG 60:1 | 0.65 | 0.02 | Glycerolipids |
| Cer 41:0;3O | 0.64 | 0.02 | Sphingolipids |

* indicates differentially abundant lipids

S8. Active and suppressed pathways based on Lipid Class

| **Active reaction** | | | **Suppressed reaction** | | |
| --- | --- | --- | --- | --- | --- |
| **Reactions chains** | **Z-score** | **Predicted genes** | **Reactions chains** | **Z-score** | **Predicted genes** |
| **Milk compared to Maternal plasma** | | | | | |
| PA->PI | 4.00 | CDS1, CDS2, CDIPT | DG->PC->PA | 4.27 | CHPT1, PLD1, PLD2 |
| LPC->LPA | 3.62 | ENPP2 | PE->PC->PA->DG | 3.67 | PEMT, PLD1, PLD2, PLPP1, PLPP2, PLPP3 |
| PC->PS->PE | 2.86 | PTDSS1, PISD |  |  |  |
| **Lamb plasma compared to Milk** | | | | | |
| DG->PC->PA | 3.45 | CHPT1, PLD1, PLD2 | PA->PI | 3.58 | CDS1, CDS2, CDIPT |
| PS->PE->PC->PA->DG | 3.19 | PISD, PEMT, PLD1, PLD2, PLPP1, PLPP2, PLPP3 | LPC->LPA | 3.56 | ENPP2 |
| PE->PC->PA->DG | 2.50 | PEMT, PLD1, PLD2, PLPP1, PLPP2, PLPP3 | PC->PS->PE | 2.22 | PTDSS1, PISD |
|  |  |  | PE->PS | 1.79 | PTDSS2 |
| **Lamb plasma compared to Maternal plasma** | | | | | |
| PA->PI | 3.64 |  | DG->PA->PG | 3.94 | CDS1, CDS2, PTPMT1 |
|  |  |  | PA->PG | 3.61 | PTDSS1 |
|  |  |  | PC->PS | 2.24 | PTDSS2 |
|  |  |  | PE->PS | 1.74 |  |
